# Diagnostic accuracy of point-of-care ultrasound with artificial intelligence-assisted assessment of left ventricular ejection fraction

**DOI:** 10.1038/s41746-023-00945-1

**Published:** 2023-10-28

**Authors:** Pouya Motazedian, Jeffrey A. Marbach, Graeme Prosperi-Porta, Simon Parlow, Pietro Di Santo, Omar Abdel-Razek, Richard Jung, William B. Bradford, Miranda Tsang, Michael Hyon, Stefano Pacifici, Sharanya Mohanty, F. Daniel Ramirez, Gordon S. Huggins, Trevor Simard, Stephanie Hon, Benjamin Hibbert

**Affiliations:** 1 CAPITAL Research Group, Division of Cardiology, University of Ottawa Heart Institute, Ottawa, Ontario, Canada; 2 Division of Cardiology, University of Ottawa Heart Institute, Ottawa, Ontario, Canada; 3 School of Epidemiology and Public Health, University of Ottawa, Ottawa, Ontario, Canada; 4 Division of Cardiology, Knight Cardiovascular Institute, Oregon Health and Sciences University, Portland, OR USA; 5 Department of Medicine, University of Ottawa, Ottawa, Ontario Canada; 6 Division of Cardiology, Tufts Medical Center and Tufts University School of Medicine, Boston, MA USA; 7 Department of Cardiovascular Medicine, Mayo Clinic, Rochester, MN USA; 8 Division of Pulmonary and Critical Care Medicine, Tufts Medical Center and Tufts University School of Medicine, Boston, MA USA

**Keywords:** Laboratory techniques and procedures, Cardiovascular diseases

## Abstract

Focused cardiac ultrasound (FoCUS) is becoming standard practice in a wide spectrum of clinical settings. There is limited data evaluating the real-world use of FoCUS with artificial intelligence (AI). Our objective was to determine the accuracy of FoCUS AI-assisted left ventricular ejection fraction (LVEF) assessment and compare its accuracy between novice and experienced users. In this prospective, multicentre study, participants requiring a transthoracic echocardiogram (TTE) were recruited to have a FoCUS done by a novice or experienced user. The AI-assisted device calculated LVEF at the bedside, which was subsequently compared to TTE. 449 participants were enrolled with 424 studies included in the final analysis. The overall intraclass coefficient was 0.904, and 0.921 in the novice (*n* = 208) and 0.845 in the experienced (*n* = 216) cohorts. There was a significant bias of 0.73% towards TTE (*p* = 0.005) with a level of agreement of 11.2%. Categorical grading of LVEF severity had excellent agreement to TTE (weighted kappa = 0.83). The area under the curve (AUC) was 0.98 for identifying an abnormal LVEF (<50%) with a sensitivity of 92.8%, specificity of 92.3%, negative predictive value (NPV) of 0.97 and a positive predictive value (PPV) of 0.83. In identifying severe dysfunction (<30%) the AUC was 0.99 with a sensitivity of 78.1%, specificity of 98.0%, NPV of 0.98 and PPV of 0.76. Here we report that FoCUS AI-assisted LVEF assessments provide highly reproducible LVEF estimations in comparison to formal TTE. This finding was consistent among senior and novice echocardiographers suggesting applicability in a variety of clinical settings.

## Introduction

Cardiovascular disease is the leading cause of mortality worldwide, with disease prevalence nearly doubling since 1990^1^. The rising prevalence of cardiac disease has dramatically increased the financial burden on healthcare systems and has further constrained access to limited resources. Transthoracic echocardiography (TTE), the most frequently utilized cardiovascular test, accounted for approximately $1.2 billion in Medicare spending in 2010, which accounted for 11% of its spending on imaging services^2,3^.

Recent technological advances in ultrasound components along with declining costs have led to the development of pocket-sized devices that are increasingly being utilized outside the confines of a formal echocardiography laboratory^4^. As a result, point-of-care ultrasound (PoCUS) is being used by clinicians from diverse clinical backgrounds as part of their cardiovascular assessment^5^, where it has been shown to outperform physical examination and improve diagnostic accuracy^6–8^.

Assessment of left ventricular ejection fraction (LVEF) is a fundamental component in the focused cardiac ultrasound (FoCUS) examination. While TTE is the standard of care to determine LVEF in clinical practice, it is often not readily available for immediate bedside evaluation and remains a scarce resource in particular communities^9^. Accordingly, the prevalence of FoCUS in clinical practice has increased to facilitate rapid clinical assessment. The reliability of FoCUS to screen for left ventricular (LV) dysfunction has been previously demonstrated^6,7^, however, in the majority of prior studies FoCUS assessment of LVEF has been limited to trained sonographers and clinicians with formal echocardiography training^6,7^. To the contrary, in real-world clinical settings, FoCUS is routinely used in primary care, anesthesia and emergency departments and are commonly performed by providers with limited PoCUS training^10^. Consequently, uncertainty regarding the accuracy of LVEF assessments and the potential impact of erroneous results on patient care remain a concern^11,12^. Due to the potential impact that FoCUS and bedside LVEF could have on patient management, it is imperative that LVEF is accurately measured by clinicians.

Artificial intelligence (AI) technologies present a potential solution to these concerns, yet insufficient validation of these novel algorithms in real-world settings limit their widespread implementation^13–15^. To date, the majority of AI-assisted LVEF assessment has been performed by echocardiographers and trained sonographers using formal TTE machines^16,17^. While early studies with FoCUS have been promising, it remains unclear whether the integration of AI into FoCUS devices improves diagnostic accuracy in real-world clinical settings where a diverse range of user background and experience exists.

Herein, we present a pragmatic prospective cohort study assessing the accuracy of AI-assisted LVEF evaluation. Our objectives were (1) to determine the accuracy of bedside AI-assisted LVEF compared to formal TTE, (2) to assess the real-world efficacy of AI-assisted LVEF assessment through comparative analysis between early and experienced scanners, and (3) to determine if AI-assisted LVEF assessments can accurately classify the severity of LV dysfunction. The hypothesis of our study is that AI-assisted LVEF assessments are a reliable index text for determining the presence of cardiac dysfunction and severity in comparison to the reference formal echocardiogram.

## Results

### Baseline characteristics

A total of 449 participants were enrolled in the study including 227 in the novice scanner (NS) subgroup and 222 in the experienced scanner (ES) subgroup. After the exclusion of studies due to non-diagnostic image quality (*n* = 25) the final cohort included 208 and 216 studies (*p* = 0.008) in the NS and ES subgroups, respectively (Supplementary Fig. 1). A formal TTE was able to calculate a LVEF in all of the excluded studies.

The median age for the overall cohort was 65 (IQR 20) years including 144 (34%) female participants (Table 1). The median BMI was 27.1 (IQR 6.8) kg/m^2^. Previously documented LV dysfunction on prior TTE was present in 101 (23.8%) participants, while 125 (29.5%) participants had LV dysfunction on their present TTE. The NS performed 92 of 125 (73.4%) abnormal studies and 29 of 33 (87.8%) with severely reduced LVEF. NS recruited from inpatient settings (emergency department, wards and intensive care units) at the University of Ottawa Heart Institute while ES recruited from the outpatient settings from Tufts Medical Centre.

**Table 1:** Baseline patient characteristics.

Characteristic	All Studies (*n* = 424)	Experienced Scanner (*n* = 216)	Novice Scanner (*n* = 208)
Age – median years (IQR)	65 (20)	62.5 (23.5)	67.5 (19.5)
Female – no. (%)	144 (34.0)	83 (38.4)	61 (29.3)
Body mass index – median kg/m^2^ (IQR)	27.1 (6.8)	27.3 (8.4)	26.7 (5.5)
Weight – median kg (IQR)	80.6 (23.9)	82.5 (23.1)	77.8 (23.5)
Hypertension – no. (%)	250 (59.0)	143 (66.2)	107 (51.4)
Dyslipidemia – no. (%)	225 (53.1)	129 (59.7)	96 (46.2)
Diabetes – no. (%)	107 (25.2)	52 (24.1)	55 (26.4)
Previously documented LV dysfunction – no. (%)	101 (23.8)	79 (36.6)	22 (10.6)
Previous CVA – no. (%)	30 (7.1)	14 (6.5)	16 (7.7)
COPD – no. (%)	28 (6.6)	19 (8.8)	9 (4.3)
Echocardiogram Characteristics			
Median LVEF – % (IQR)	56.1 (14.8)	60.0 (10.9)	52.0 (18.4)
Normal LVEF	299 (70.5)	183 (84.7)	116 (55.8)
Abnormal LVEF	125 (29.5)	33 (15.3)	92 (44.2)
Mild dysfunction	56 (13.2)	16 (7.4)	40 (19.2)
Moderate Dysfunction	36 (8.5)	13 (6.0)	23 (11.1)
Severe Dysfunction	33 (7.8)	4 (1.9)	29 (13.9)

### LVEF assessment

**Fig. 1:**
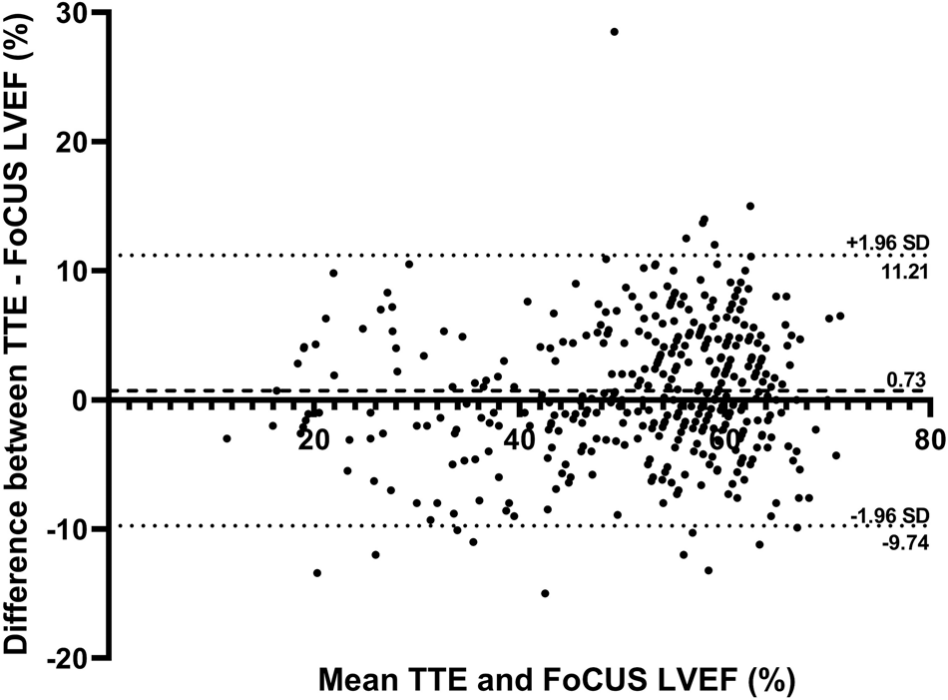
Bland-Altman plot for LVEF assessment by bedside AI-assisted FoCUS and TTE.

The ICC for the entire cohort showed excellent reliability with a value of 0.904. The NS and ES subgroups have excellent and good reliability with ICC values of 0.921 and 0.845, respectively. The Bland-Altman plot shows a significant bias of 0.73% towards TTE (*p* = 0.005) with a level of agreement of 11.2% (Fig. 1). Simple linear regression demonstrated a correlation between bedside AI-assisted LVEF and TTE LVEF (R^2^ of 0.82, root mean squared error [RMSE] 5.33, mean absolute error [MAE] 4.25, *p* < 0.0001; Supplementary Figure 2A). The correlation between bedside AI-assisted LVEF and TTE LVEF assessment in both the NS (R^2^ = 0.85, RMSE 5.31, MAE 3.86, *p* < 0.0001; Supplementary Figure 2B) and ES (*R*^2^ = 0.72, RMSE 5.23, MAE 4.48, *p* < 0.0001; Supplementary Figure 2C) subgroups.

The ability to correctly classify LVEF severity by bedside AI-assisted FoCUS was compared to TTE LVEF (Table 2). In cases where there was disagreement between the AI-assisted LVEF and TTE LVEF classification, only two cases (0.5%) disagreed by more than one severity category. This corresponded to a Cohen’s weighted kappa of 0.83 (confidence interval [CI] 0.76–0.91).

**Table 2:** Comparison in the classification of LVEF severity between bedside AI-assisted FoCUS and TTE.

Reference LVEF	Automated AI assessment			
	Normal (≥50%)	Mildly Reduced LVEF (40–49.9%)	Moderately Reduced (30–39.9%)	Severely Reduced LVEF (≤30%)
Normal (≥50%)	276	22	1	0
Mildly Reduced LVEF (40–49.9%)	8	45	3	0
Moderately Reduced (30–39.9%)	1	7	21	7
Severely Reduced LVEF (≤30%)	0	0	8	25

**Fig. 2:**
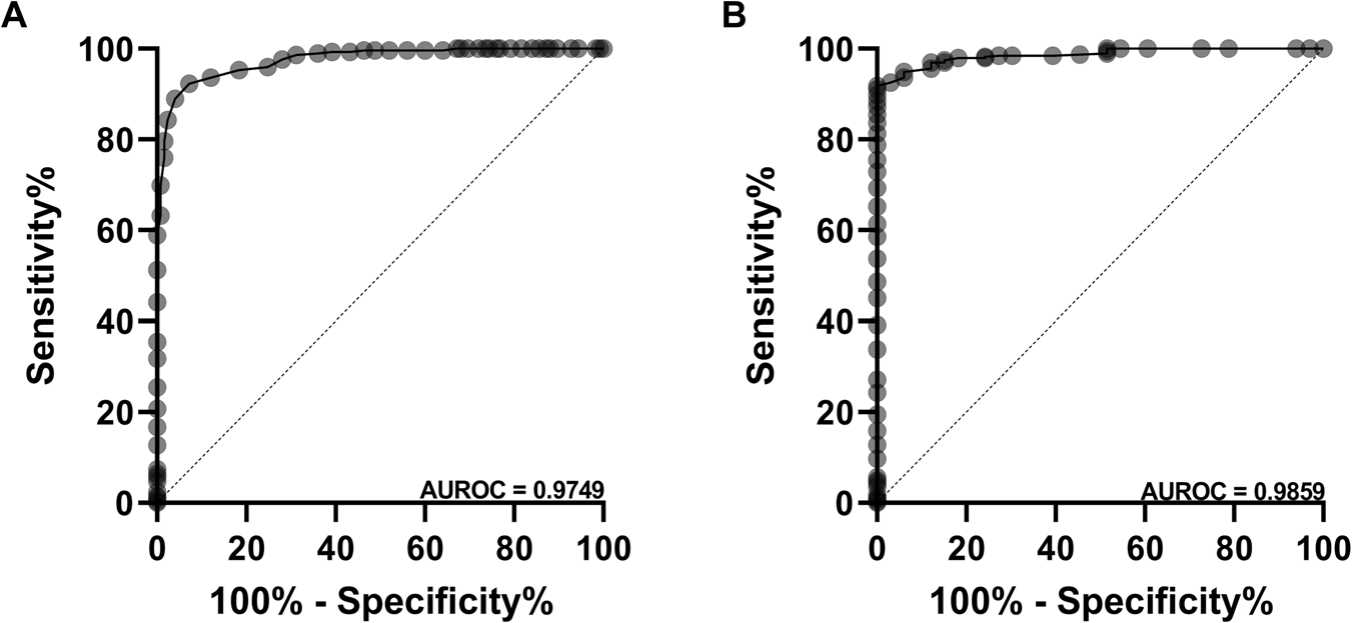
AUROC curves for the diagnosis of LV dysfunction. Diagnostic performance ofbedside AI-assisted FoCUS for identification of **A**) abnormal (<50%) and **B**) severely reducedLVEF (<30%).

ROC analysis for the detection of an abnormal LVEF (<50%) by bedside AI-assisted FoCUS had an area under the curve (AUC) of 0.98 (CI 0.96–0.99; Fig. 2A), which correlates to a sensitivity of 92.8% (CI 86.4–96.4), specificity of 92.3% (CI 88.5–95.0), NPV of 0.97 (CI 0.94–0.98) and PPV of 0.83 (CI 0.76–0.89). In ROC analysis, the detection of severe LV dysfunction (LVEF < 30%) by AI-assisted FoCUS had an AUC of 0.99 (CI 0.98–1.00; Fig. 2B) with a corresponding sensitivity of 78.1% (CI 59.6–90.1), specificity of 98.0% (CI 95.9–99.0), NPV of 0.98 (CI 0.96–0.99) and PPV of 0.76 (CI 0.57–0.88). Cohort analysis demonstrates a weighted kappa of 0.83 (CI 0.77–0.88) and 0.80 (0.72–0.88) in the NS and ES groups, respectively.

## Discussion

In this prospective, multicentre, observational cohort study of 424 participants we found that bedside FoCUS with AI-assisted LVEF assessment has diagnostic performance when compared to comprehensive TTE read by a board-certified echocardiographer. Independent of the level of experience of the FoCUS scanner, users can accurately identify the presence and severity of LV dysfunction with high degrees of certainty. These findings suggest that in both inpatient and outpatient settings, AI-assisted FoCUS assessments may serve as a surrogate for a formal TTE in order to assess LVEF.

The assessment of LVEF by FoCUS has mostly been studied without AI, with image acquisition and interpretation completed independently by the bedside user. In a meta-analysis on the diagnostic accuracy of FoCUS, an abnormal LVEF has been reported to be identified with a sensitivity and specificity of 84% and 89%, respectively^6^. Comparatively, the majority of these studies had an experienced sonographer or echocardiographer as the bedside user^11,18–20^. In the remaining two studies that used novice FoCUS operators, the sensitivity and specificity for an abnormal LVEF (defined as less than 50%) was 85–86% and 82–89%, respectively^21,22^. Furthermore, there are only two studies that classified the severity of LV dysfunction, with both studies using experienced operators^18,19^. While pivotal to the growth of FoCUS, there are methodologic concerns that limit the applicability of these studies to clinical practice. The clinical FoCUS user population is diverse in terms training and experience and is unlike the highly subspecialized user population in these studies. As a result, the documented diagnostic accuracy of non-AI LVEF assessment may not be reflective of the real-world application. Despite its importance, there is a paucity in evidence outlining whether severity can be accurately identified at the bedside outside the hands of trained echocardiographers. The integration of AI into FoCUS remains a potential method for minimally trained users to accurately and efficiently identify abnormal LVEF, while simultaneously providing a reliable evaluation of severity.

Integration of AI has been mostly studied within general echocardiography but emerging data has shown promise in FoCUS^23–25^. A recent validation study of 100 participants demonstrated that FoCUS AI-assisted LVEF assessments could be accurately done with a correlation coefficient of 0.87 and a sensitivity and specificity of 90% and 87% for LVEF less than 50%, respectively^26^. Admittedly, these images were acquired by an experienced echocardiographer and in an optimal setting of a formal echo laboratory, but nevertheless these results demonstrate the potential of AI-assisted FoCUS assessment in real-world practice. Open-source AI-LVEF software are now becoming available; an emergency department study of EchoNet-POCUS achieved an AUROC of 0.81 (0.78–0.85) for identifying reduced LV function. A notable difference in model training is the use of physician visual assessment rather than quantitative methods like the biplane method of disks. While the current iteration does not comment on the severity of dysfunction, this is a promising noncommercial alternative^27^. Beyond these studies, our study provides robust evidence by providing multicenter data across a spectrum of users and clinical context.

The current study, which represents the largest cohort of patients evaluated using AI-assisted FoCUS, builds upon the previous data by validating the utility of AI-assisted LVEF using a design that is reflective of real-world clinical practice. First, we have shown that AI can reliably and accurately identify LV dysfunction. The current standard for FoCUS is a visual assessment for LVEF which may be more susceptible to misclassification^11,12^. As a result of our findings, AI-assisted LVEF assessments may provide a safety measure to confirm the bedside clinician’s impression, while also providing the degree of severity. Given the potential changes to management depending on the degree of LV severity, AI-FoCUS may provide a rapid, accurate assessment of LVEF and therefore allow for prompt diagnosis and management. Second, we have shown that LVEF can be accurately calculated with a novice user using the AI-assisted FoCUS device. FoCUS examinations may be deferred by clinicians due to the lack of training, comfort, or clinical expertise and as a result bedside ultrasound is only used in two to five percent of emergency department assessments^28,29^. The novice users in this study, who did not have formal FoCUS or echocardiography training, demonstrates that inexperienced users can use AI-FoCUS to accurately determine LVEF. This avails LVEF assessment to a broader and more comprehensive spectrum of users than traditional FoCUS such as regions with limited cardiac and sonography infrastructure^30,31^.

With the concern surrounding the implementation of AI into real-world clinical care, our study has specific methodological strengths that highlight its applicability into current practice^13–15^. Our study has a pragmatic design that is based on real patient encounters. Participants were not excluded based on their clinical presentation or setting, body habitus or cardiac rhythm. Image acquisition was often done with participants in less-than-ideal positioning, including supine or sitting upright related to their presenting disease. Despite these challenges, 94.4% and 91.6% of all and NS studies were diagnostic quality. As a result, the limitations that commonly impact FoCUS in real-world practice were incorporated in the study design, meaning that our results are reflective of current clinical practice. Additionally, this is a large multicentre, international study that integrates the differences in image acquisition between centres and countries.

Interestingly, the diagnostic accuracy for LVEF AI-assessments were better with NS in comparison to ES. Considerations for this discrepancy include heterogeneity due to differences in the primary recruitment settings between cohorts and nonrandom distribution of cases. The ES subgroup had less studies excluded from the data analyses which likely reflects their ability to obtain studies in patients with challenging imaging windows but this could be confounded by the difference between the primary recruitment settings between the groups. Potentially, the images are less likely to be diagnostic quality and are often challenging to interpret and have likely impacted the findings in ES^32^. Our study also finds a lower error between AI and echocardiographers compared to independent cardiology assessments^33^. We hypothesize that this likely due to several factors including the differences from variability due to expertise and reader-fatigue, as well minimizing cognitive biases present in human diagnosis^34^.

Nonetheless, there are important limitations to our study. Due to participant enrollment being completed as a convenience series, there could be selection bias to exclude critically unwell patients that needed a timely assessment by an experienced sonographer. Additionally, the findings of our study are specific to the AI technology of the EchoNous KOSMOS device and performance might not be duplicated with similar but different platforms. Furthermore, it is important to highlight that this AI technology only provides assistance in interpretation and not in image acquisition; this is reflected with the differences in diagnostic quality images between the ES and NS cohorts. With new and upcoming AI software providing for assistance in image acquisition, our findings are only limited to image interpretation^35^. Another limitation of our study is the heterogeneity between the NS and ES groups. The NS group recruited mostly inpatients from one centre while the ES group mostly recruited outpatients from the other centre. This may be a confounder in the difference the number of diagnostic quality studies between cohorts. Finally, while TTE read by trained echocardiographers was utilized as the gold standard – inter-observer variability can introduce error in the comparative gold standard *and* better modalities, such as MRI, can be used for precise LVEF assessment.

In summary, artificial intelligence-assisted FoCUS performed by both novice and experienced scanners can accurately determine LVEF compared to a comprehensive TTE.

## Methods

### Study design

**Fig. 3:**
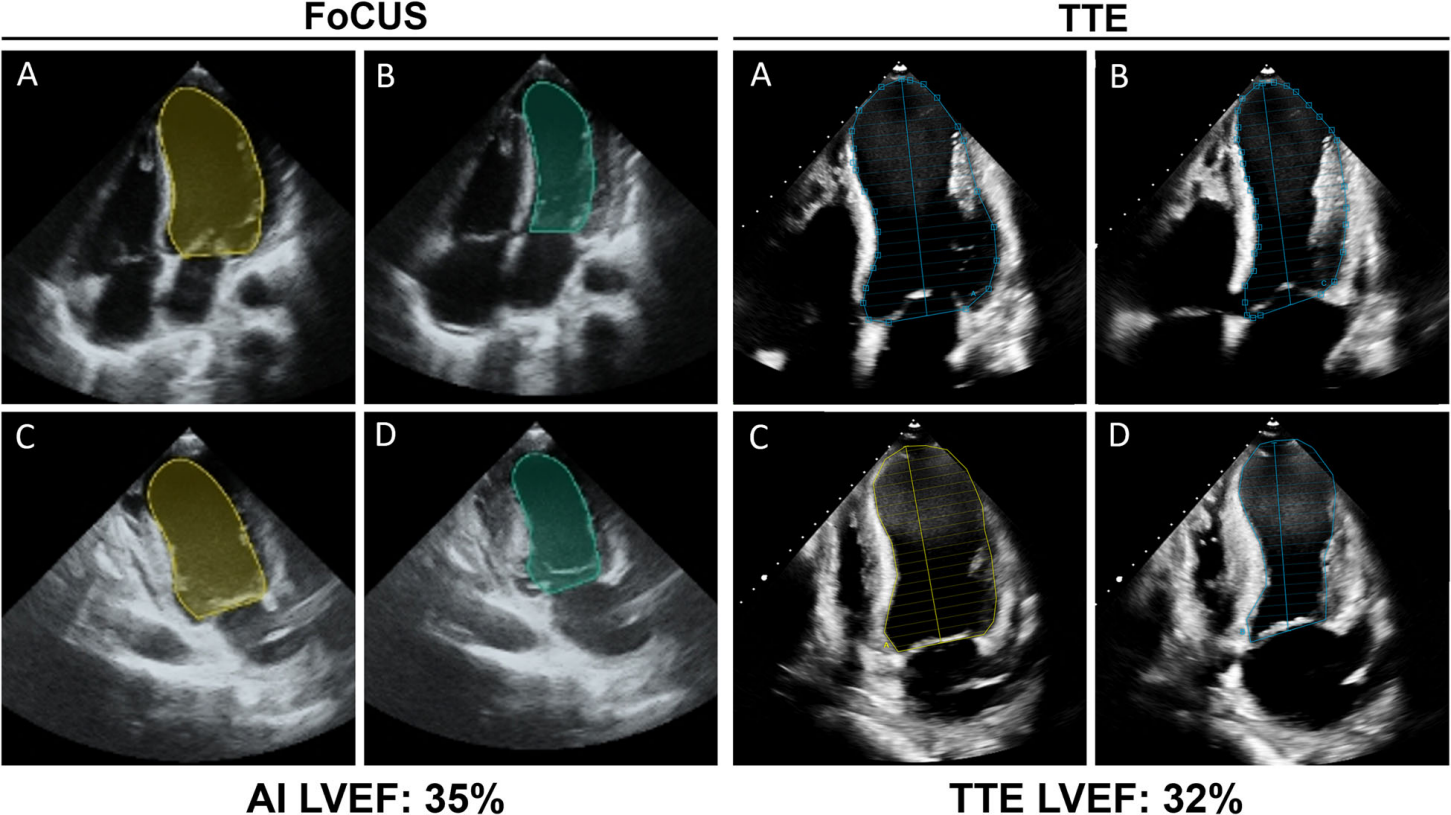
AI-assisted LVEF FoCUS and TTE image acquisition. LV cavity tracings by automated AI and manually by an echocardiographer of the A4C end-diastolic (**A**) and –systolic (**B**) frames and A2C end-diastolic (**C**) and –systolic (**D**) frames to calculate LVEF.

This was a prospective, multicentre, observational cohort study conducted at The Ottawa Hospital (Ottawa, Canada), University of Ottawa Heart Institute (Ottawa, Canada) and Tufts Medical Centre (Boston, United States). All adult (≥18 years of age) patients undergoing TTE as part of their routine clinical care between December 2020 and March 2022 were eligible for study inclusion. Participants were enrolled in a convenience series from inpatient (critical care, ward, and emergency department) and outpatient settings. FoCUS assessment of LVEF was performed within 48 h of TTE image acquisition (Fig. 3). There were no exclusion criteria. All participants, or their substitute decision maker, provided informed verbal consent prior to enrollment. Ethics approval was obtained from the Ottawa Health Science Network Research Ethics Board and Tufts Institutional Review Board (Supplementary Document).

Participants had their FoCUS evaluation completed by either a novice or experienced scanner. The novice scanners were fellows meeting competency for FoCUS that had completed less than 100 scans. The experienced scanners included trained cardiac sonographers. We included the experienced scanners to act as a control group to help understand whether differences between AI LVEF and cardiologist-quantified LVEF in novice doing scans in real-life clinical settings was related to image acquisition from experience or the technology itself. The EchoNous KOSMOS point-of-care ultrasound machine device (Redmond, United States) was used for all FoCUS examinations by both novice and experienced scanners. Image acquisition was completed with participants laying in the left lateral decubitus position, and if unable, modified images with the patient supine or sitting upright was done. During bedside AI-assisted assessment, the study frames and LV tracings completed by the AI were left unmodified. Studies were excluded from the data analysis if the images were non-diagnostic, and the device was unable to calculate a LVEF.

### AI device description, development and assisted assessments

The EchoNous KOSMOS point-of-care ultrasound machine device (Redmond, United States) was used for the AI-assisted LVEF assessments. KOSMOS is a hand-held, 64-channel diagnostic medical ultrasound system that uses machine-learning workflow to facilitate cardiac image acquisition and estimate LVEF. The model used in this study included a bridge tablet, where images could be reviewed and edited, and a torso probe which was used for image acquisition. The AI algorithm calculates LVEF using the modified Simpson’s biplane method of disks. The device was internally validated with a study of over 1200 patients from inpatient wards, coronary care units and emergency departments as well as during cardiology consultation. A subgroup of 100 patients were used to validate the AI-assisted LVEF assessments, which found a correlation of 0.87 (*p* < 0.0001) with cart-based machines (https://echonous.com/clinical-benchmarking-kosmos-platform/). The device has received 510(k) U.S. Food and Drug Administration (FDA) clearance. For the purposes of this study, the AI was used only for image interpretation and not for image acquisition.

**Fig. 4:**
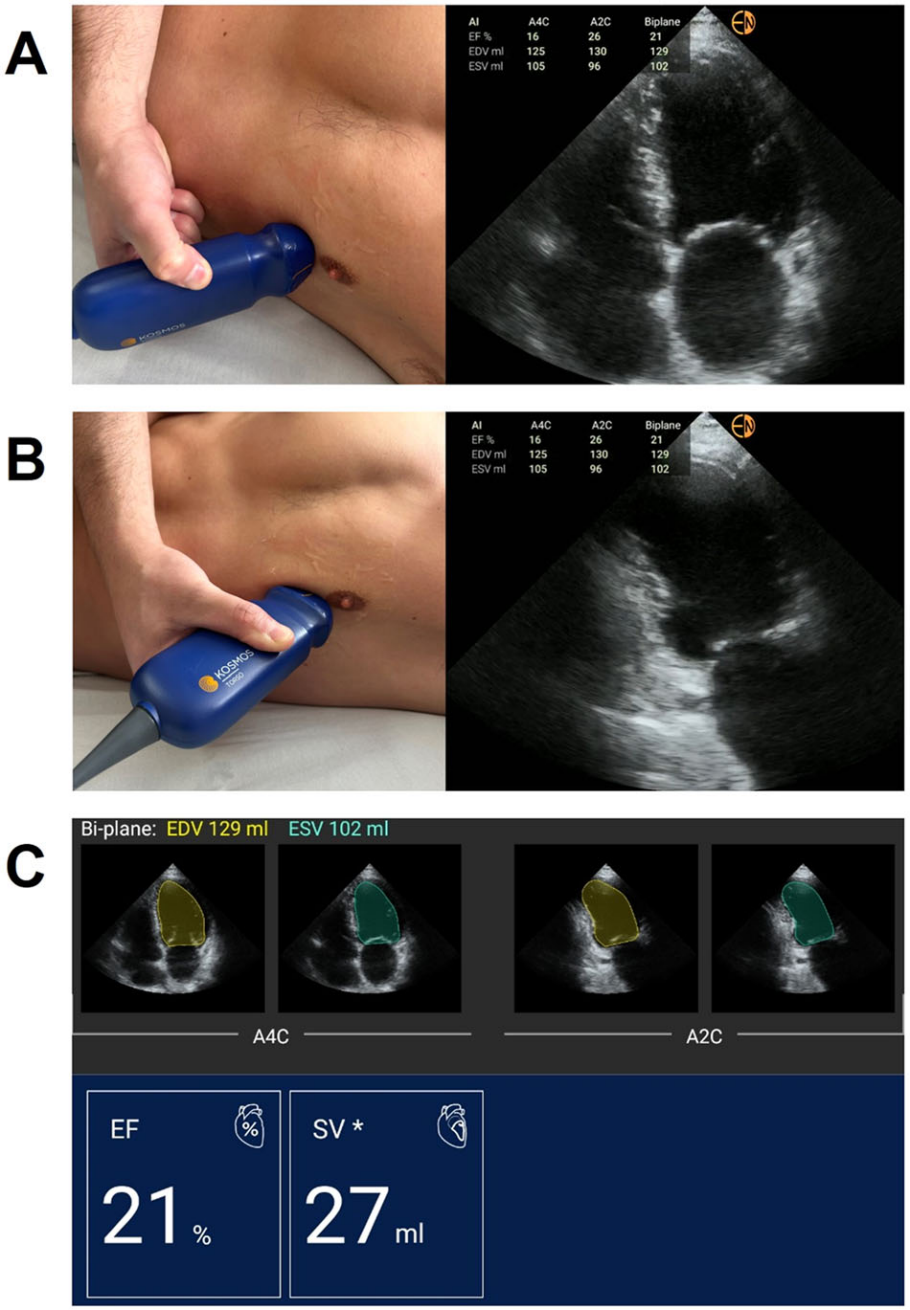
The AI-assisted LVEF workflow using FoCUS. **A** Step 1: acquire five-second clip ofA4C view, **B** Step 2: acquire five-second clip of A2C view, **C** Step 3: AI identifies enddiastolic and -systolic frames, traces endocardial border and calculates LVEF.

The AI workflow begins with the prospective acquisition of a five-second recording of the apical four-chamber view then the apical two-chamber view. The device automatically identifies the end-diastolic and end-systolic frames, automatically traces the left ventricular (LV) endocardial border and calculates the LVEF using the biplane method of disks (Fig. 4, Supplementary Video). If image acquisition was not possible with the participant laying in the left lateral decubitus position, modified images with the patient supine or sitting upright was done. Of note, the end-diastolic and -systolic frames, and LV tracing can be modified by the user, though as previously noted, were left unmodified for the purposes of this study.

### Transthoracic echocardiography assessment of left ventricular ejection fraction

All participants underwent a comprehensive TTE following the recommendations by American Society of Echocardiography^18^. Image acquisition for all TTE studies was completed by a trained cardiac sonographer using a cart-based system and interpretation was performed by an experienced level-three echocardiographer. The LVEF was determined using the biplane method of disks by manually tracing the end-diastolic and end-systolic LV endocardial borders in the A4C and A2C views.

### Statistical analysis

Continuous variables were summarized using means and standard deviations (SD) if normal distribution or with median and interquartile range (IQR) if non-normally distributed. Categorical variables were summarized using frequencies and percentages. All non-diagnostic studies were excluded from our data analyses.

Agreement between the bedside AI-assisted and TTE LVEF was analyzed using simple linear regression and intraclass correlation (ICC) of the combined cohort and within the novice and experienced scanner subgroups. A Bland-Altman plot was performed, and the level of agreement and bias was calculated between AI-assisted and TTE LVEF. LVEF was categorized as normal (≥50%), mild (40–49.9%), moderate (30–40%) or severe dysfunction (<30%). The LVEF from the comprehensive TTE study was used as the standard reference. Categorical agreeability was calculated with a Cohen’s weighted kappa coefficient. The performance of bedside AI-assisted LVEF assessment for identification of abnormal (<50%) and severely reduced LVEF was assessed using a receiver operating characteristic (ROC) analysis. The sensitivity, specificity, positive predictive value (PPV) and negative predictive value (NPV) for abnormal and severely reduced LVEF were determined. All reported *p* values are two-sided, and a value less than 0.05 was considered to indicate statistical significance. Analyses were performed using SAS software, version 9.4 (SAS Institute, North Carolina, USA). Our findings were presented as per the STARD reporting guidelines. Devices used in this study for obtaining bedside AI-assisted LVEF were provided by Echonous.

### Reporting summary

Further information on research design is available in the Nature Research Reporting Summary linked to this article.

### Supplementary information

Supplementary Materials

Supplementary Document

Supplementary Video.

Reporting Summary

## Data Availability

Due to the strict ethical approvals provided in this study, the data used in this study is available from the corresponding author upon request and after establishment of data sharing agreement between institutions.

## Code availability

Due to the strict ethical approvals provided in this study, code for all data analysis is available from the corresponding author upon request and establishment of data sharing agreement between institutions.
