# Supplementary material for: Diagnostic accuracy of point-of-care ultrasound with artificial intelligence-assisted assessment of left ventricular ejection fraction
Source: NPJ Digit Med. 2023 Oct 28;6:201. doi: 10.1038/s41746-023-00945-1 (PMC10613290; doi:10.1038/s41746-023-00945-1)
Supplement: Supplementary file 1 — Supplementary Materials [file 41746_2023_945_MOESM1_ESM.docx]

**Supplementary Materials: Diagnostic accuracy of point-of-care ultrasound with artificial intelligence-assisted assessment of left ventricular ejection fraction**

Pouya Motazedian MD, Jeffrey A Marbach MBBS MS, Graeme Prosperi-Porta MD MSc, Simon Parlow MD, Pietro Di Santo MD, Omar Abdel-Razek MD, Richard Jung MD PhD, William B Bradford, Miranda Tsang, Michael Hyon, Stefano Pacifici MD, Sharanya Mohanty MD, F. Daniel Ramirez, Gordon S Huggins MD, Trevor Simard MD, Stephanie Hon MD and Benjamin Hibbert MD PhD


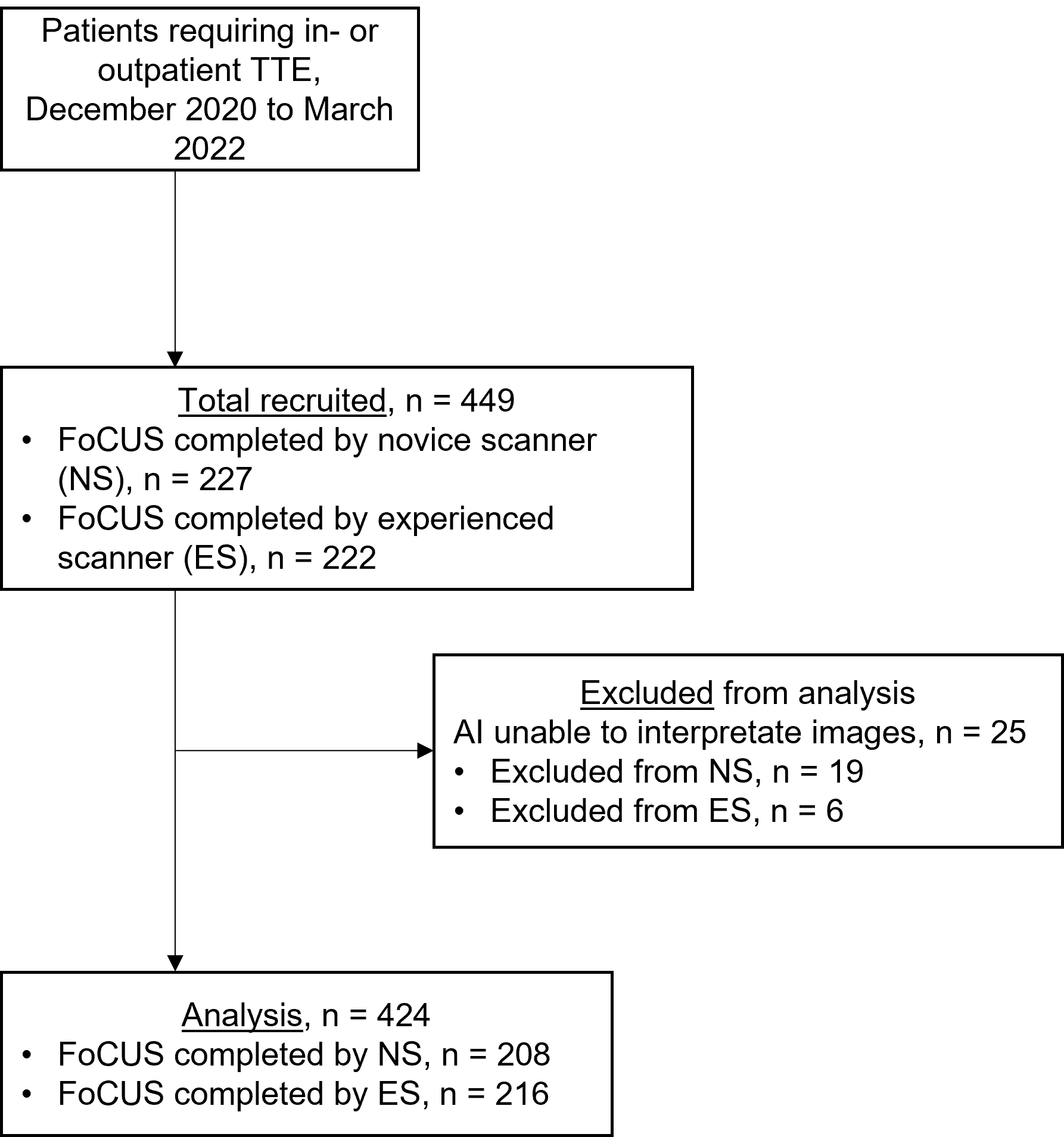


Supplementary Figure 1. STROBE Study flow diagram.


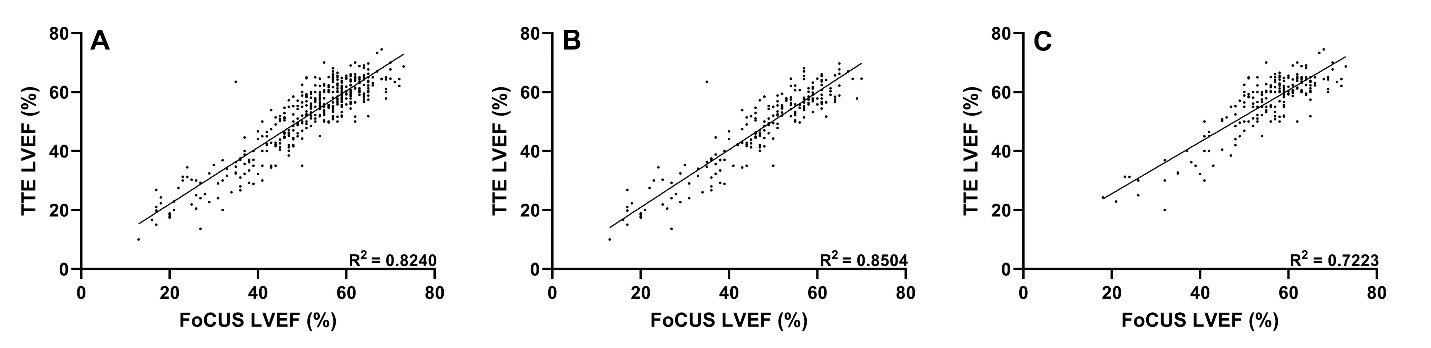


Supplementary Figure 2. Simple linear regression model between bedside AI-assisted FoCUS and TTE LVEF for all diagnostic studies (A), novice scanner studies (B) and experienced scanner studies (C).
