# Supplementary material for: Diagnostic accuracy of point-of-care ultrasound with artificial intelligence-assisted assessment of left ventricular ejection fraction
Source: NPJ Digit Med. 2023 Oct 28;6:201. doi: 10.1038/s41746-023-00945-1 (PMC10613290; doi:10.1038/s41746-023-00945-1)
Supplement: Supplementary file 2 — Supplementary Document [file 41746_2023_945_MOESM2_ESM.docx]

**STUDY TITLE:** Assessment of Left Ventricular Systolic Function with Focused Cardiac Ultrasound: Validation of Automated Ejection Fraction, Stroke Volume, and Cardiac Output Detection

**STUDY SPONSOR:** Dr. Gordon Huggins

**PRINCIPAL INVESTIGATOR**

Dr. Gordon Huggins, MD

Associate Professor of Medicine

Division of Cardiology, Department of Medicine

Tufts Medical Center and Tufts School of Medicine

800 Washington Street, Tupper Bldg. 11^th^ Floor

Boston, MA, 02111

617-636-2807

Ghuggins@tuftsmedicalcenter.org

**VERSION DATE: 11.29.21**

**Table of Contents** – Click on the links below to go directly to the applicable section

[A. Study Schema 3](#_Toc89082597)

[B. Introduction 3](#_Toc89082598)

[B.1 Background and Rationale 3](#_Toc89082599)

[B.2 Risks to Subjects 4](#_Toc89082600)

[B.3 Potential Benefits to Subjects 4](#_Toc89082601)

[B.4 Alternatives 4](#_Toc89082602)

[C. Objectives 5](#_Toc89082603)

[D. Enrollment and Withdrawal 5](#_Toc89082604)

[D.1 Inclusion Criteria 5](#_Toc89082605)

[D.2 Exclusion Criteria 5](#_Toc89082606)

[D.3 Withdrawal of Subjects 5](#_Toc89082607)

[D.4 Recruitment and Retention 6](#_Toc89082608)

[D.4.1 Local Recruitment Methods 6](#_Toc89082609)

[D.4.2 Study-Wide Recruitment Methods 6](#_Toc89082610)

[D.4.3 Payment 6](#_Toc89082611)

[D.4.4 Reimbursement 6](#_Toc89082612)

[E. Costs to Subjects 7](#_Toc89082613)

[F. Study Design 7](#_Toc89082614)

[F.1 Study Timelines 7](#_Toc89082615)

[F.2 Procedures 7](#_Toc89082616)

[F.3 Evaluations 9](#_Toc89082617)

[F.4 Collection and Storage of Human Biological Specimens (Tissue Banking) 9](#_Toc89082618)

[G. Ethics and Protection of Human Subjects 9](#_Toc89082619)

[G.1 Informed Consent Process 9](#_Toc89082620)

[G.2 Waiver or Alteration of Consent Process 10](#_Toc89082621)

[G.3 International Research 10](#_Toc89082622)

[G.4 Confidentiality 10](#_Toc89082623)

[G.5 Screening Data Collection Form/Screening Log 11](#_Toc89082624)

[G.6 Provisions to Protect the Privacy Interests of Subjects 12](#_Toc89082625)

[G.7 Provisions to Monitor the Study to Ensure the Safety of Subjects 12](#_Toc89082626)

[G.8 Vulnerable Populations 13](#_Toc89082627)

[H. Adverse Event Monitoring 14](#_Toc89082628)

[H.1 Definitions 14](#_Toc89082629)

[H.2 Reporting Procedures 14](#_Toc89082630)

[H.3 Reportable New Information 14](#_Toc89082631)

[I. Statistical Considerations 14](#_Toc89082632)

[I.1 Study Endpoints 14](#_Toc89082633)

[I.2 Sample Size Justification Statistical Analyses 14](#_Toc89082634)

[I.3 Number of Subjects 15](#_Toc89082635)

[I.4 Data Management 15](#_Toc89082636)

[I.5 Randomization 15](#_Toc89082637)

[J. Drugs or Devices 15](#_Toc89082638)

[K. Study Administration 16](#_Toc89082639)

[K.1 Setting 16](#_Toc89082640)

[K.2 Registration 16](#_Toc89082641)

[K.3 Resources Available 16](#_Toc89082642)

[K.4 IRB Review 17](#_Toc89082643)

[K.5 Multi-Site Research 17](#_Toc89082644)

[K.6 Community-Based Participatory Research 17](#_Toc89082645)

[K.7 Sharing Results with Subjects 17](#_Toc89082646)

[L. References 17](#_Toc89082647)

[M. Protocol Ammendment 18](#_Toc89082648)

# Study Schema
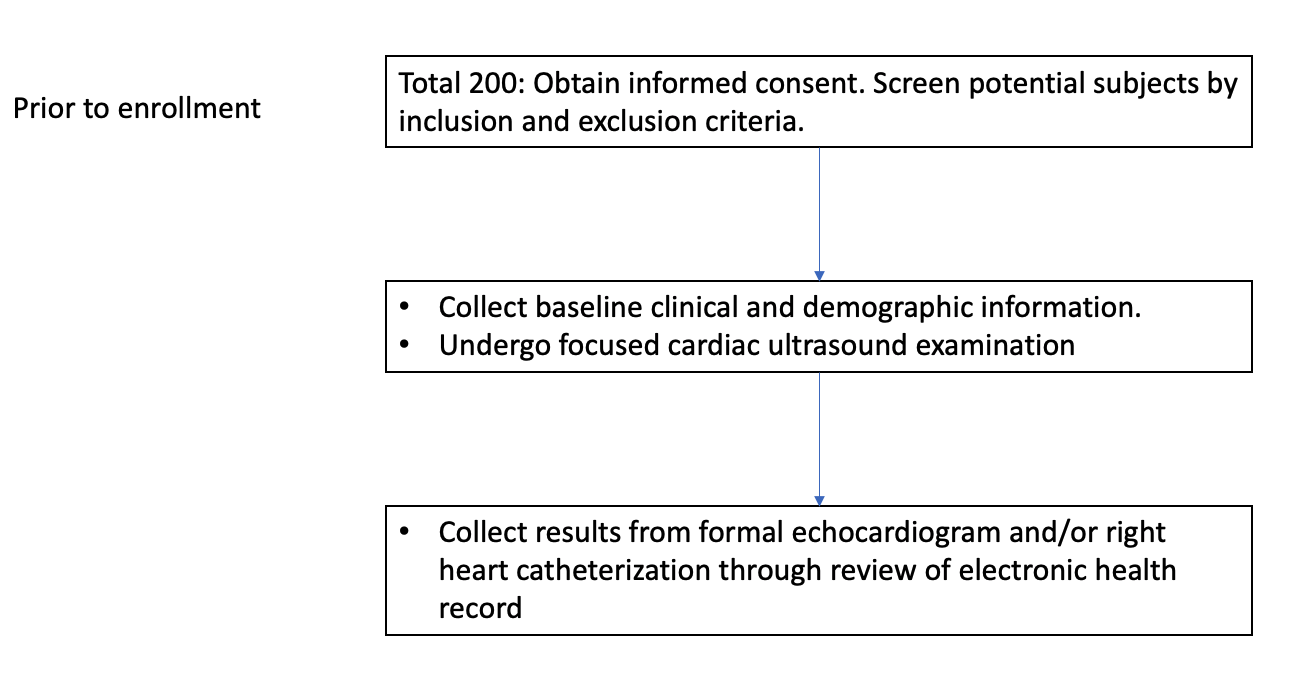


# Introduction

##

## B.1 Background and Rationale

The role of ultrasound in bedside patient assessment has expanded drastically over the past two-decades. In particular, technological advances in recent years – which have considerably reduced the size and cost of ultrasound devices – have expanded the availability of ultrasound beyond the confines of formal diagnostic laboratories.^1–3^ Due to the significant difficulty, and unreliability, of cardiovascular assessment, bedside cardiac ultrasound – coined focused cardiac ultrasound (FoCUS) – is increasingly being incorporated into the routine evaluation for cardiac pathology.^3,4^

While several societal guidelines have outlined the recommended elements of a FoCUS assessment, perhaps none is most important than evaluation of left ventricle (LV) systolic function.^5–8^ Formal evaluation of LV systolic function, with transthoracic echocardiography (TTE), is assessed through measuring the difference between the end-diastolic and end-systolic volumes of the LV, and dividing by the end-diastolic volume – thereby calculating the left ventricular ejection fraction (LVEF).^9^ Calculating LV volumes through 2D linear measurements (i.e. Teichholz and Quinones methods) are not recommended due to the potential for significant inaccuracy as a result of the geometric assumptions required with these methods. Rather, the American Society of Echocardiography recommends that, when using 2D echocardiography, LV volumes are measured using the biplane method of disks summation (modified Simpson’s rule), whereby the LV cavity is traced in the apical four- and apical two-chamber views at end-systole and end-diastole.^9^ These volume measurements can then be applied, as described above, to calculate the LVEF, as well as to estimate the stroke volume and cardiac output. Hemodynamic parameters such as stroke volume and cardiac output can also be obtained from invasive right heart catheterizations. During a right heart catheterization, pressure measurements are obtained via a pulmonary artery catheter inserted via the right jugular vein.

While the modified Simpson’s rule is the ideal method for evaluation of LVEF from 2D images, this has been either not possible (i.e. small screen sizes and inability to perform frame-by-frame measurements) or not practical (i.e. insufficient training) when performing a FoCUS assessment. As a result, to date, we have relied on visual LVEF evaluation as part of the FoCUS examination. Although there is evidence of good correlation between visual estimation of LVEF, this is highly dependent on the level of training and expertise of the individual capturing and interpreting the images and vulnerable to subjective bias.^2^

To overcome this obvious limitation there has been significant interest in automating the assessment of LV systolic function through incorporation of artificial intelligence (AI) into point-of-care ultrasound devices. Several devices with AI have received FDA and Health Canada approval and early studies have demonstrated very good correlation between non-experts assessment of LVEF with that of experts.^10^ Although these early results are promising, there have been no adequately powered, real-world, clinical studies assessing the accuracy of AI LVEF detection.

Presently, the attending physicians, fellows, and residents at Tufts Medical Center have been using the KOSMOS (EchoNous, Redmon, WA) portable ultrasound device to perform FoCUS assessments as part of routine patient care, including utilization of the automated LVEF, stroke volume, and cardiac output features. This information is incorporated into the overall clinical diagnosis and decision-making process, much like the information obtained from the patient’s history and physical examination.

- 1. This is not the first time the study drug, device, or intervention/procedure will be used in humans
  2. Is there an active control group?

**Yes  No**

If **Yes**, respond to all of the following:

1. *Check to confirm that* *the active control is an established effective intervention. If it is not, clarify how it is ethically justified to use this control in the study:*
2. *Describe any potential bias in the selection of the active control such that there will be an unfair advantage for the investigational intervention. For example, is the active control treatment known to be significantly less effective in this study population than another treatment:*       or

N/A

1. *Check to confirm that the sample size and the randomization ratio for this active control study is ethically justified with regard to the number of participants who will be exposed to the risks of the study.*

## B.2 Risks to Subjects

- 1. List the reasonably foreseeable risks, discomforts, hazards, and/or inconveniences to the subjects related to their participation in the research, including risk of unintentional loss of confidentiality. Include a description of the probability, magnitude, duration, reversibility, and potential consequences of the risks. Consider physical, psychological, social, legal, and economic risks:
- This study involves no more than minimal risks, as the study intervention is a non-invasive FDA-approved ultrasound examination. Records identifying study participants will be kept confidential and, to the extent permitted by the applicable laws, will not be disclosed or made publicly available, except as described in the consent document. De-identified data from this study may be used for future research purposes. Even though the likelihood that someone may identify participants from the study data is very small, it can never be completely eliminated – therefore there is a minor risk of loss of confidentiality.
  1. State which study interventions may have unknown risks:       or N/A
  2. State which study interventions may have risks to an embryo or fetus (if a subject is or becomes pregnant) or to a nursing infant of a study subject:       or N/A
  3. Describe risks to people other than the participating subject, e.g., risks to family members, friends, others or risks to the community:       or  N/A
  4. Are there any risks to study investigators or staff performing the study procedures due to research with high risk populations (e.g. prisoners, intravenous drug users, patients with major psychiatric issues, etc.):?

**Yes  No**

If **Yes**, respond to all of the following:

- 1. *Describe these risks:*
  2. *Describe the procedures that will be put in place to minimize these risks:*
  3. *Describe how these procedures are adequate for the location where study procedures will be performed:*
  4. *Check to confirm that you have contacted research administration to clarify whether additional approvals (e.g. Security, Risk Management, etc.) are needed for the performance of this study with high risk populations.*

## B.3 Potential Benefits to Subjects

- 1. Describe the potential benefits that individual subjects may experience from taking part in the research. Include the probability, magnitude, and duration of the potential benefits:
  2. Check if there is no direct benefit.
  3. Describe any benefit to the population from which the subject is drawn:
- We hope the information learned from this study will help us determine whether the results obtained from a portable ultrasound are reliable, and can, possibly, replace the need for obtaining a formal echocardiogram and/or right heart catheterization for some patients in the future.
  1. Describe any benefit to science, society, and humanity in general:       or  NA

##

## B.4 Alternatives

1. Describe alternatives to participating in this research study (e.g. to decide not participate in the study, alternative treatments, no treatment (palliative care), etc.):

Participation in this research is completely voluntary. The alternative to participating is to not participate. In either case all participants will receive standard of care medical treatments.

1. Describe the standard clinical care that may be an alternative:       or  N/A
2. Describe how the subject can receive the research procedures/drug/device used in this study in a non-research setting:       or  N/A

# Objectives

1. Describe the purpose, specific aims, or objectives of the study (i.e. the reason for performing the study in terms of the scientific question to be answered):

The aim of this study is to evaluate the accuracy of the KOSMOS portable ultrasound automated LVEF, stroke volume, and cardiac output detection in a real-world, clinical setting. We hypothesize that automated results will demonstrate a strong correlation with formal TTE and right heart catheterization studies.

- The primary outcome of interest is the overall diagnostic accuracy of the automated LVEF, stroke volume, and cardiac output computation as compared to either TTE (for LVEF assessments) or right heart catheterization (for stroke volume and cardiac output assessments).
- The secondary outcome of interest is time to LVEF acquisition with FoCUS

#

# Enrollment and Withdrawal

## D.1 Inclusion Criteria

- 1. Describe the criteria that define who will be **included** in the study as a numbered list:
- All patients who are cared for at Tufts Medical Center and:
  - Provide informed consent
  - Age greater than or equal to 18 years old
  - Undergo bedside FoCUS examination with the KOSMOS portable ultrasound device
  - Have had a TTE within 30 days, or have had a TTE ordered by the primary treating team and/or have had/are scheduled for a right heart catheterization with calculation of stroke volume and cardiac output

##

## D.2 Exclusion Criteria

- 1. Describe the criteria that define who will be **excluded** in the study as a numbered list:
- Unwillingness or inability to provide informed consent by the patient or substitute decision maker for healthcare decisions
- Patients with known, moderate or greater, regurgitant valvular disease (i.e. aortic regurgitation, mitral regurgitation, tricuspid regurgitation).

1. Describe in detail how the eligibility criteria will be assessed and satisfied (e.g., medical record review, physical examination):

Medical record review and discussion with patient

1. State who will determine eligibility. Note that those who are designated to determine eligibility must have appropriate training, expertise, and oversight, for example a physician PI or Co-I on a biomedical study:

- The primary and co-investigators (i.e. Drs. Huggins, Marbach, and Hon). .

1. *Can study subjects participate in another research study while participating in this research study:*

Yes  No

## D.3 Withdrawal of Subjects

1. *Describe anticipated circumstances under which subjects will be withdrawn from the research without their consent:*

As the only intervention in this research study is the performance of a bedside cardiac ultrasound, which will take place as soon as possible after consent has been obtained, we do not anticipate any subjects being withdrawn from the research without their consent. If for some reason the patient’s primary treating physician determines that it is in the patient’s best interests to be withdrawn from the study then they will need to notify they study team, at which point the patient will be removed from the study.

1. *Describe procedures that will be followed when subjects withdraw or are withdrawn from the research, including the possibility of partial withdrawal from study intervention with continued data collection:*

If a participant is withdrawn from the study or chooses to stop being in the study, it will not affect their care or treatment outside this study. Any data collected prior to withdrawal will still be used for the study.

1. *Describe any necessary safety precautions to be applied to subjects who withdraw or are withdrawn (tapering drug doses, evaluative x-ray, etc.):*       or N/A

##

## D.4 Recruitment and Retention

###

### D.4.1 Local Recruitment Methods

Describe the following attributes of the recruitment plan for the local Tufts site:

1. When, where, and how potential subjects will be recruited:

Potential subjects will be screened for potential recruitment by the study investigators This will occur when patients are admitted to the inpatient medical, surgical or intensive care units at Tufts Medical Center and/or when they present to TMC for an echocardiogram and/or right heart catheterization. Once screened for possible inclusion the study team will approach the patient for inclusion in the study.

1. Source of subjects (for example, patient population, local community, etc.): Subjects presenting to TMC for an echocardiogram and/or right heart catheterization and those admitted to TMC.
2. Methods that will be used to identify potential subjects: Physician referral and chart review
3. If print and media advertisements will be used, specify when, where, how long and frequency of the advertisements that will be published/aired:       or  N/A
   1. Check to confirm that any necessary permission will be obtained for posting/airing these (for example, permission to post a flyer on a bulletin board).
4. If recruitment material is being mailed or otherwise distributed, submit the proposed material and describe where/how the distribution list will be obtained:       or  N/A
5. Describe how the recruitment methods described will be effective in attracting the targeted subject population: The target population is patients who recently underwent or are scheduled to undergo an echocardiogram and/or right heart catheterization. By screening patients scheduled for these tests we will be able to efficiently identify potential subjects for recruitment.

### D.4.2 Study-Wide Recruitment Methods

Is this is a multicenter study where subjects will be recruited by methods not under the control of the local Tufts site (e.g., call centers, national advertisements)?

**Yes  No**

If **Yes**, respond to all of the following:

### D.4.3 Payment

Will subjects receive money, gifts, or any other incentive for participating in this study?

This does not include reimbursement for expenses, which is considered in the next section.

**Yes  No**

If **Yes**, respond to all of the following:

### D.4.4 Reimbursement

Will subjects be reimbursed for their expenses, such as travel, parking, meals, or any other study related costs?

**Yes  No**

If **Yes**, respond to all of the following:

1. What qualifies for reimbursement and whether pre-approval is needed:
2. How subjects will be required to document expenses for reimbursement (e.g., provide receipts):
3. How and how often subjects may request reimbursement:
4. How reimbursement will be made (e.g., cash, check, Greenphire ClinCard). Please note, if Greenphire ClinCard will not be used to provide reimbursement, you must use a log to track reimbursements to subjects, as these reimbursements are subject to audit:
5. The reimbursement schedule (when will subjects be reimbursed):

# Costs to Subjects

Does the research involve any costs to subjects?

**Yes  No**

If **Yes**, describe any costs that subjects might be responsible for due to participation in the research:

#

# Study Design

## F.1 Study Timelines

- 1. Describe the duration of an individual subject’s participation in the study:

Participants will be required to allow a Focused cardiac ultrasound be performed at the time of enrollment. Following this study there is no further requirement from the subject. The research team will follow up on the results of the subjects formal echocardiogram and/or right heart catheterization by reviewing the patient’s medical records if it is not available at the time of enrollment. After this information has been collected the subject’s participation will cease.

- 1. Describe the duration anticipated to enroll all study subjects at the Tufts study site:

12 months

- 1. Describe the estimated date for investigators to complete this study (complete primary analyses):

December 2021.

## F.2 Procedures

1. If **Yes**, respond to all of the following:
2. *Describe the scientific, methodological, and medical reasons to use a placebo:*
3. *Describe the care that will be given to subjects who receive placebo. If this is local standard of care, specify that:*
4. *Describe all potential risks to the placebo group and how the risks will be minimized.*
5. *Summarize the research design and sequentially identify all procedures to be performed to accomplish the specific aims of the project. Clearly identify and distinguish procedures that are considered experimental, procedures that are performed exclusively for research purposes (including “extra” routine tests), and procedures that would occur regardless of the research (i.e., standard of care). Point out any procedures, situations, or materials that may be hazardous, and the precautions to be exercised to maintain subject safety:*

This is a prospective cohort study. Consecutive patients cared for at Tufts Medical Center, who have had a recent (within 30 days), or are scheduled to have a subsequent TTE performed at the discretion of the treating team will be screened for participation. Also, patients who are undergoing a right heart catheterization for a clinical indication will also be eligible for recruitment.

We will prospectively collect baseline clinical and demographic characteristics at the time of enrollment. We will be accessing the electronic medical record (Sorian) and entering the information collected into RedCap. There will be no collection of data beyond the date of enrollment, with the exception of those patients whose TTE or right heart catheterization has yet to be performed, in which case the subsequent TTE/right heart catheterization report will be the only information collected prospectively beyond the day of enrollment. The specific procedures to be followed will be:

- Patients who have had a recent TTE (within 30 days), or are scheduled to have a TTE and/or right heart catheterization performed for clinical reasons are eligible to participate in this study.
- If agreeable to participate in the study then participants will receive the usual standard of care (right heart cath and/or TTE) as dictated by the treating medical team.
- Upon signing the consent form, the research team will begin to collect baseline clinical and demographic information about participants from them and/or their medical chart.
- Participants will then undergo the KOSMOS ultrasound evaluation at the time of enrollment or the earliest convenient time after enrolling in the study.
- The TTE and/or right heart catheterization data will be collected and compared with the results of the KOSMOS ultrasound.

*Please also describe the following concerning procedures:*

- - - - 1. How individuals will be screened for eligibility. Specify screening that will take place prior to informed consent and screening that will take place after informed consent:

Screening will begin through identifying individuals who are scheduled to undergo transthoracic echocardiogram and/or right heart catheterization at Tufts medical center. Patients who have recently underwent an echocardiogram may also be included if they are identified by the study team as potentially eligible for participation. There will be no additional screening prior to consent. Following consent the patient’s medical history will be evaluated by questioning the patient and, when necessary, reviewing the patient’s medical records for exclusion criteria. If no exclusion criteria are identified at this point then the patient will begin the protocol as described above.

- - - - 1. Procedures being performed to monitor subjects for safety or to minimize risks:

All data collected will be documented in the case report form in RedCap, which is stored on the Tufts secure server.

- - - - 1. All drugs and devices used in the research, their regulatory approval status, and the purpose of their use:

The KOSMOS device used in this project is an FDA approved ultrasound device that is available for commercial sale. It will be used to perform the bedside cardiac ultrasound as discussed above.

- - - - 1. The source records that will be used to collect data about subjects. (Attach all surveys, scripts, and data collection forms.):

See attached RedCap CRF

1. Is there a placebo control arm?

**Yes  No**

Describe the following concerning pregnancy testing and birth control:

- - 1. What type of pregnancy testing and how frequently will be conducted on women of reproductive potential. If testing will not be conducted provide the reason: NA
    2. What birth control methods **women** of reproductive potential will be instructed to use. If women will not be instructed about acceptable methods of birth control, clarify why: NA
    3. What birth control methods **men** of reproductive potential will be instructed to use. If men will not be instructed about acceptable methods of birth control, clarify why: NA
  1. Describe the data that will be collected during the study and how the data will be obtained:

Following identification of the study cohort (as described above), clinical data will be abstracted from the electronic medical record (Sorian) including: demographic characteristics (i.e. sex, age, height, weight, body mass index), cardiac risk factors (i.e. hypertension, diabetes, smoking, hyperlipidemia, previous myocardial infarction, previous cardiac intervention, previous strokes, heart failure, etc.), past medical history, indication for FoCUS assessment and TTE.

- - 1. If there are plans for long-term follow-up (once all research related procedures are complete), describe the data will be collected during this period:       or  N/A
  1. Specify which tests are routinely performed for clinical care, but are providing data for the research, and which tests are only performed for research purposes:

All of the formal echocardiogram and right heart catheterization tests are performed for clinical care. The bedside cardiac ultrasound is also performed to guide clinical care, primarily through its ability to provide real-time results at the time of patient evaluation. However, in the case of participants who present for an outpatient right heart catheterization and/or echocardiogram, the bedside ultrasound would not be performed for clinical care, rather, in this group of participants, the bedside ultrasound would be performed specifically for research purposes.

- 1. For Humanitarian Use Device (HUD) uses provide a description of the device, a summary of how you propose to use the device, including a description of any screening procedures, the HUD procedure, and any patient follow-up visits, tests or procedures.      or  N/A

## F.3 Evaluations

Will you perform any laboratory tests for this study?

**Yes  No**

## F.4 Collection and Storage of Human Biological Specimens (Tissue Banking)

Will biological specimens be stored for **future, unspecified**, research?

**Yes  No**

If **Yes**, respond to all of the following:

1. Where specimens will be stored, how long they will be stored, how specimens will be accessed, and who will have access to the specimens:
2. List the Protected Health Information (PHI) to be stored or associated with each specimen. If PHI will be gathered in the future and associated with the specimen, describe the frequency of gathering such PHI:       or  N/A, PHI will not be stored or associated with each specimen.
3. Procedures to release specimens, including: the process to request a release, approvals required for release, who can obtain specimens, and the PHI to be provided with specimens:
4. Risks to subjects and their families associated with this collection and storage for future research use:
5. Risks to groups or populations associated with this collection and storage for future research use:
6. The mechanism by which the research subject can withdraw permission to use the stored specimens and associated PHI for future research. Indicate what will happen to the specimens and related research data if permission is withdrawn:

Refer to the IRB’s [Policy for Research Involving Collection and Storage of Human Biological Specimens for Future Research](https://viceprovost.tufts.edu/tissue-banking-policy) to ensure that all relevant information is included in the protocol.

# Ethics and Protection of Human Subjects

##

## G.1 Informed Consent Process

Will subjects be required to provide informed consent?

**Yes  No**

If **Yes**, respond to all of the following:

- 1. Where the consent process will take place (e.g. a private clinic room): In a private clinic or inpatient hospital room.
  2. Anticipated amount of time a potential subject will have to make a decision about participation in the study: 30 minutes
  3. Processes to ensure ongoing consent throughout the study: If the subject changes their mind after giving consent and withdraws consent the study procedure will stop immediately. During the brief period of time when the participant is actively engaged in the research process (during the scan principally) if the study personnel become aware of a change in the subject’s capacity to give consent or to participate in study procedures the study procedure will stop.
  4. Role of each research team member involved in the informed consent process (Please note, in general, for studies designated by the IRB as greater than minimal risk, a physician PI or Co-I must perform the informed consent process with subjects. A study coordinator/clinical research associate may not consent subjects. A study coordinator/clinical research associate may assist with this process; however, the PI or Co-I should be present to discuss the study with the subject and answer questions, and the PI or Co-I should sign the ICF documenting that s/he has performed the informed consent process with the subject and the subject understands the study.): The informed consent process will be undertaken by the PI orCo-Investigators.
  5. Check to confirm you will follow “[SOP: Informed Consent Process for Research (HRP-090)](https://eirb.tuftsmedicalcenter.org/IRB/sd/Doc/0/GFRQ9IFG3FGKVB96RUV3Q41F9F/HRP-090%20-%20SOP%20-%20Informed%20Consent%20Process%20for%20Research_2.4.pdf)”.

If **not**, answer all of the following:

- 1. Steps that will be taken to minimize the possibility of coercion or undue influence:
  2. Steps that will be taken to ensure subjects’ understanding:
  3. Describe in detail any other changes that differ from the SOP:
  4. *Check to confirm that Non-English speakers will be enrolled using interpreters and IRB approved Short Forms per the* [*IRB’s Short Form policy*](http://viceprovost.tufts.edu/HSCIRB/irb-regulations/short-form-consent-documents-and-translations-into-commonly-encountered-languages/). *If IRB approved Short Forms will not be used, describe which languages the consent will be fully translated into, who will conduct the consent interview, use of interpreters, use of IRB approved translated documents, etc.:*
  5. *If non-English speakers are not eligible (excluded from enrollment) for this study, provide the ethical and scientific justification, including whether this would be equitable. For example, if non-English speakers are eligible for the study and could potentially benefit from participation, it would not be equitable to exclude them:* N/A

Refer to the [IRB Short Form Policy](http://viceprovost.tufts.edu/HSCIRB/irb-regulations/short-form-consent-documents-and-translations-into-commonly-encountered-languages/) on the IRB website for information about enrolling non-English speakers including how to reach medical interpreters.

1. *Check to confirm you will follow “*[*SOP: Written Documentation of Consent (HRP-091)*](https://eirb.tuftsmedicalcenter.org/IRB/sd/Doc/0/UL3H8IJ6BNOKLBPKKE9OBC1Q6E/HRP-091%20-%20SOP%20-%20Written%20Documentation%20of%20Consent_2.4.pdf)*”. If* ***not****, describe how consent will be documented in writing:*
2. *Check to confirm you will follow “*[*SOP: Remote Consent Process (HRP-092)*](https://eirb.tuftsmedicalcenter.org/IRB/sd/Doc/0/PHLGATBIDA8KBCI2E0JIAMN22B/HRP-092%20-%20SOP%20-%20Remote%20Consent%20Process_060820.pdf)*” if there is ever a situation where consent will* ***not*** *be obtained in person. If you will follow a different process if there is ever a situation where consent will* ***not*** *be obtained in person, describe:*       or  N/A*.*

If **Yes**, respond to all of the following:

- 1. Where the consent process will take place (e.g. a private clinic room):
  2. Anticipated amount of time a potential subject will have to make a decision about participation in the study:
  3. Processes to ensure ongoing consent throughout the study:
  4. Role of each research team member involved in the informed consent process (please note, for a biomedical study, a physician PI or Co-I should perform the informed consent process with subjects. A study coordinator may assist with this process; however, the PI or Co-I should be present to discuss the study with the subject and answer any questions, and the PI or Co-I should sign the ICF documenting that s/he has performed the informed consent process with the subject):
  5. Check to confirm you will follow “[SOP: Informed Consent Process for Research (HRP-090)](http://viceprovost.tufts.edu/HSCIRB/files/HRP-090_SOP_Informed-Consent-Process-Research.pdf)”.

If **not**, answer all of the following:

- 1. Steps that will be taken to minimize the possibility of coercion or undue influence:
  2. Steps that will be taken to ensure subjects’ understanding:
  3. *Check to confirm that Non-English speakers will be enrolled using interpreters and IRB approved Short Forms per the* [*IRB’s Short Form policy*](http://viceprovost.tufts.edu/HSCIRB/irb-regulations/short-form-consent-documents-and-translations-into-commonly-encountered-languages/). *If IRB approved Short Forms will not be used, describe which languages the consent will be fully translated into, who will conduct the consent interview, use of interpreters, use of IRB approved translated documents, etc.:*
  4. *If non-English speakers are not eligible (excluded from enrollment) for this study, provide the ethical and scientific justification, including whether this would be equitable. For example, if non-English speakers are eligible for the study and could potentially benefit from participation, it would not be equitable to exclude them:*

Refer to the [IRB Short Form Policy](http://viceprovost.tufts.edu/HSCIRB/irb-regulations/short-form-consent-documents-and-translations-into-commonly-encountered-languages/) on the IRB website for information about enrolling non-English speakers including how to reach medical interpreters.

1. *Check to confirm you will follow “*[*SOP: Written Documentation of Consent (HRP-091)*](http://viceprovost.tufts.edu/HSCIRB/files/HRP-091_SOP_Written-Documentation-Consent.pdf)*”. If* ***not****, describe how consent will be documented in writing:*
2. *Check to confirm you will follow “*[*SOP: Remote Consent Process (HRP-092)*](https://viceprovost.tufts.edu/HSIRB/files/HRP-092-RemoteConsentProcess.pdf)*” if there is ever a situation where consent will* ***not*** *be obtained in person. If you will follow a different process if there is ever a situation where consent will* ***not*** *be obtained in person, describe:*       or  N/A*.*

## G.2 Waiver or Alteration of Consent Process

This applies for studies where informed consent will not be obtained, required information will not be disclosed, or the research involves deception.

1. Is a waiver or alteration of the consent process being requested for this study?

**Yes  No**

If **Yes**, respond to all of the following:

1. *Provide the rationale for the waiver:* In order to identify patients eligible for enrollment the study team will need to determine which patients are scheduled to undergo an echocardiogram and/or right heart catheterization. To determine this the study investigators will need to be able to review the list of patients scheduled for these investigations.
2. *How the waiver or consent alteration will* ***NOT*** *adversely affect the rights and welfare of subjects:* The investigators will only review the patients to determine eligibility. They will not review any clinical or demographic data apart from that necessary to determine eligibility.
3. *How the research could* ***NOT*** *practicably be carried out without the waiver or alteration:* Without the waiver of consent the research team would not have a viable method of identifying partipicants eligible for enrollment in the study.
4. *How, subjects will be provided with additional pertinent information after participation. If subjects will not be provided this information after participation, explain why:*  The patients deemed eligible for enrollment will be approached by the investigators to request study participation. All details pertaining to the study will be provided at that time and informed consent will be performed prior to inclusion in the study.

If **Yes**, respond to all of the following:

1. *Provide the rationale for the waiver:*
2. *How the waiver or consent alteration will* ***NOT*** *adversely affect the rights and welfare of subjects:*
3. *How the research could* ***NOT*** *practicably be carried out without the waiver or alteration:*
4. *How, subjects will be provided with additional pertinent information after participation. If subjects will not be provided this information after participation, explain why:*
   1. Is a waiver of the consent process being requested for parents for research involving children?

**Yes  No**

If **Yes**, respond to all of the following:

- 1. Is a waiver of the consent process for planned emergency research being requested?

**Yes  No**

If **Yes**, respond to all of the following:

1. *How the subjects are in a life-threatening situation:*
2. *How available treatments are unproven or unsatisfactory*:
3. *How the collection of valid scientific evidence, which may include evidence obtained through randomized placebo-controlled investigations, is necessary to determine the safety and effectiveness of particular interventions:*
4. *Clarify how the appropriate animal and other preclinical studies that have been conducted, and the information derived from those studies and related evidence that supports the potential for the study drug, device, or procedure to provide a direct benefit to the individual subject:*
5. *How the research could not practicably be carried out without the waiver:*
6. *How obtaining informed consent is not feasible because:*
7. *Subjects will not be able to give their informed consent as a result of their medical condition (describe why):*
8. *The study drug, device, or procedure must be administered before consent from the subjects’ legally authorized representatives can be obtained (describe why):*
9. *There is no reasonable way to identify prospectively the individuals likely to become eligible for participation in the research (describe why):*
10. *The length of the potential therapeutic window based on scientific evidence:*
11. *Check to confirm that the research team will attempt to contact a legally authorized representative for each subject within that window of time and, if feasible, ask for consent within that window rather than proceeding without consent. Note that at continuing review, you will need to report to the IRB the efforts made to contact legally authorized representatives or subject’s family member if a legally authorized representative is not available.*
12. *Check to confirm that if the subject dies before a legally authorized representative or family member can be contacted, information about the research will be provided to the subject’s legally authorized representative or family member, if feasible.*
13. *Check to confirm that if a legally authorized representative or family member is told about the research and the subject’s condition improves, the subject will also to be informed as soon as feasible.*
14. *The consultation (including, where appropriate, consultation carried out by the IRB) with representatives of the communities in which the research will be conducted and from which the subjects will be drawn:*
15. *The public disclosure to the communities in which the research will be conducted and from which the subjects will be drawn, prior to initiation of the research, of the plans for the investigation and its risks and expected benefits:*
16. *The public disclosure following completion of the research to apprise the community and researchers of the study, including the demographic characteristics of the research population, and its results:*
17. *The procedures to inform, at the earliest feasible opportunity, each subject, or if the subject remains incapacitated, a legally authorized representative of the subject, or if such a representative is not reasonably available, a family member, of the subject’s inclusion in the research, the details of the investigation and other information contained in the ICF, and that he or she may discontinue the subject’s participation at any time without penalty or loss of benefits to which the subject is otherwise entitled:*

## G.3 International Research

*Refer to the IRB’s* [*International Research Checklist*](https://eirb.tuftsmedicalcenter.org/IRB/sd/Doc/0/4FUMCVJNRQF4T54J3AVET8NU33/International%20Research%20Checklist_071519.docx) *and* [*International Research Guidance*](https://eirb.tuftsmedicalcenter.org/IRB/sd/Doc/0/48BQMF8656K4P76QU0ERKMDL6A/International%20Research%20Guidance_Downloaded%20from%20IRB%20website_09.06.19.docx) *and include all relevant information described in those documents in this protocol:*

## G.4 Confidentiality

Data and specimens should be stored in a secure location only accessible by the research team. The PI must ensure that study documents are stored in a manner that protects the privacy of subjects and the confidentiality of study data.

1. *State where the study records, both electronic and/or paper documents including signed ICFs/assent forms, will be retained during the study (state the location for original document plus any copies that are made, e.g., if a copy of the ICF will be retained in the subject’s medical record):*

The Principal Investigator will maintain adequate and accurate records to enable the conduct of the study to be fully documented and the study data to be subsequently verified. These documents will be classified into two separate categories: [1] Investigator's Study File and [2] patient clinical source documents. The Investigator's Study File will contain the protocol/amendments, CRF and schedule of assessments, Independent Ethics Committee/Institutional Review Board approval with correspondence, sample informed consent, and authorization forms and other appropriate documents/correspondence, etc.

Patient clinical source documents will include patient medical records, signed informed consent forms. All personal health information collected will be kept confidential unless release is required by law. For audit purposes only, the Tufts Health Sciences Institutional Review Board and Tufts Medical Center may review the patients’ medical records under the supervision of Dr. Gordon Huggins and his staff. All paper records will be stored in a locked filing cabinet and/or office at Tufts Medical Center. All electronic records with subject identifiers will be stored on a Redcap database that is password protected, only accessible by Dr. Gordon Huggins and the co-investigators. All data extracted from the RedCap database will be de-identified and coded with a unique ID for each subject and will be held in a password protected file stored on a hospital server. No identifiable information will be stored on any mobile devices (laptops, USB keys, CDS, DVDs, etc).

1. *State where study records will be retained when the study has been closed (long-term storage):*

The Investigator will keep source documents as described above on file for 10 years after completion or discontinuation of the study. After that period of time the documents may be destroyed, according to local regulations. After the analysis is completed (expected timeframe less than 2-years) deidentified data will be extracted from RedCap and then the study deleted from RedCap.

1. *State who, in addition to the research team, will have access to the study files, data, and/or specimens:* NA
2. *Will data (or specimens) be sent outside of Tufts Medical Center or Tufts University* ***and/or sent between Tufts Medical Center and Tufts University****?*

**Yes  No**

## G.5 Screening Data Collection Form/Screening Log

This section specifically refers to data collected about potential subjects who are screened, but have not signed consent, for example potential subjects whose medical record is reviewed to see if they are potentially eligible, potential subjects who respond to a telephone screening call where the research team records information about the potential subject, etc. In this section “Screening Data / Screening Log” refers to any form of data collection on potential subjects who have not yet signed consent. For more information, refer to <https://privacyruleandresearch.nih.gov/clin_research.asp>.

Will a screening data/screening log be used in this research study?

**Yes  No**

If **Yes**, respond to all of the following:

1. *Check to confirm you have submitted the Screening Data Collection Form / Screening Log to the IRB.*
2. *Review the following and provide information about the Screening Data / Screening Log and how it will be used (****check all that apply****):*
   1. *De-identified Screening Log will be provided to and/or viewed by the Study Sponsor (the log does not record any* [*HIPAA identifiers*](http://viceprovost.tufts.edu/HSCIRB/policies-regulations/hipaa/definitions/) *or contain protected health information (PHI)).*
      1. *Explain how the Screening Log will be “distributed to” or viewed by the study Sponsor, i.e., how the Screening Log be e-mailed, sent to and /or viewed by the study Sponsor:*
   2. *Identifiable Screening Log that will* ***not*** *be distributed or viewed outside of the institution (although the Screening Log will record* [*HIPAA identifiers*](http://viceprovost.tufts.edu/HSCIRB/policies-regulations/hipaa/definitions/)*, the Screening Log will* ***not*** *leave the institution.)*
      1. *Specify the identifiers that will be collected (e.g. date of admission or clinic visit, medical record #, and reason the person was not eligible for the study):*
   3. *Identifiable Screening Log that will be distributed or viewed outside of the institution.*

*Consider whether these identifiers could be removed from the Screening Log. It might be possible to eliminate HIPAA identifiers or use a screened subject identifier code and maintain a separate key to the code so no PHI will leave the institution.*

*If it is necessary to include HIPAA identifiers in the screening log, address the following:*

*The rationale for including HIPAA identifiers in the Screening Log:*

*A plan for protecting the privacy and confidentiality of screened subjects. Screened subjects might or might not be enrolled in the research study, and since screened subjects will not have consented to the use of their PHI for research purposes, it is especially important to protect their privacy and confidentiality. The plan should include keeping identifiers to a minimum, and keeping the Screening Log in a secure location (password protected computer location only accessible by the research team or a locked file cabinet in a locked office, only accessible by the research team):*

Note: A Screening Data Collection Form / Screening Log that contains identifiers and/or PHI must NOT be sent to the study Sponsor UNLESS the IRB has granted a waiver of consent/authorization for this component of the study (or unless the investigator has obtained IRB approved consent and research authorization from each study subject whose name is on the log.)

## G.6 Provisions to Protect the Privacy Interests of Subjects

In this section, “privacy” refers to persons and their interest in controlling the access of others to themselves. For example, based on their privacy interests, people want to control:

- The time and place where they give information
- The nature of the information they give
- The nature of the experiences that are given to them
- Who receives and can use the information

Persons might not want to be seen entering a place that might stigmatize them, such as a pregnancy counseling center that is clearly identified as such by signs on the front of the building. What is private depends on the individual and can vary according to gender, ethnicity, age, socioeconomic class, education, ability level, social or verbal skill, health status, legal status, nationality, intelligence, personality, and the individual’s relationship to the researcher. Protecting the privacy interests of a young child might mean having a parent present at a session with a researcher. Protecting the privacy interests of a teenager might mean having a parent absent.

- 1. Describe the steps that will be taken to protect subjects’ privacy interests (e.g. ensuring that discussion of the study will take place in a private area where subjects cannot be overheard):

Discussion of the study will take place in in a private area where subjects cannot be overheard. All information collected throughout the study will be kept strictly confidential. As above, all data will be entered into and stored in the Tufts RedCap database, including a unique ID number for each subject. When data is extracted from the database for statistical analysis it will be coded by the unique ID and not include identifiable information. The extracted data will be entered into a spreadsheet, which will be password protected and stored on a secure hospital server. The only means of connecting the de-identified data extracted from the RedCap database to the patient identifiers will be through access to both the RedCap database and the secure, password protected spreadsheet. These will both only be accessible to the investigator and co-investigators. No identifiable information will be stored on any mobile devices. There will be no patient interventions performed for this study. All data will be kept and stored securely as per regulations.

- 1. Describe the steps that will be taken to make the subjects feel at ease with the research situation in terms of the questions being asked and the procedures being performed. “At ease” does not refer to physical discomfort, but the sense of intrusiveness a subject might or might not experience in response to questions, examinations, and procedures (e.g. ensuring that subjects are comfortable with the research team members performing the study procedures):

During the process of obtaining consent we will provide as much time as is needed to answer questions and to ensure that the subject has as much time as is required to make an informed decision to participate in this study. The actual engagement in research activities is about 30 minutes and we will continue to answer questions and to make the subject feel at ease throughout the entire research process.

## G.7 Provisions to Monitor the Study to Ensure the Safety of Subjects

1. *Describe the plan to periodically evaluate the data regarding both harms and benefits to assess subject safety as follows:*
   1. *The data that will be reviewed, including safety data, untoward events, and efficacy data:* NA. There is no planned safety data as the intervention is a non-invasive study that will not affect the participants clinical care. No interim analysis is planned for assessment of efficacy.
   2. *Who will review the data:* NA
   3. *How the safety information will be obtained and documented (e.g., case report forms, by telephone calls with participants, printouts of laboratory results, etc.):* NA
   4. *The frequency of data collection, including when safety data collection starts:* NA
   5. *The frequency or periodicity of review of cumulative data:* NA
   6. *The statistical tests for analyzing the safety data to determine whether harm is occurring:* NA
   7. *Any conditions that trigger an immediate suspension of the research or other action for the research:* NA

The plan might include establishing a data monitoring committee which addresses all the above.

1. *Describe the entity responsible for monitoring the data, and their respective roles (e.g., the investigators, the research sponsor, a coordinating or statistical center, an independent medical monitor, a Data and Safety Monitoring Board (DSMB) /Data Monitoring Committee (DMC), and/or some other entity, and the timeframe for reporting events to this entity:* NA
2. *A copy of the DSMB/DMC Charter if the study is enclosed with the submission:* Yes N/A

## G.8 Vulnerable Populations

If the research involves individuals who are vulnerable to coercion or undue influence, describe the rationale for their inclusion and the additional safeguards included to protect their rights and welfare.

- 1. Can or will pregnant women be enrolled?

**Yes  No**

- 1. Can or will the research involve neonates of uncertain viability or non-viable neonates?

**Yes  No**

If **Yes**, respond to all of the following:

1. Any preclinical and clinical studies that have been conducted that provide data for assessing potential risks to neonates:
2. The important biomedical knowledge that will be developed from this research and why it cannot be obtained by other means:
3. Whether there will be added risk to the neonate resulting from the research:
4. How individuals providing consent are informed of the reasonably foreseeable impact of the research on the neonate:
5. No person shall perform or offer to perform an abortion where part or all of the consideration for said performance is that the fetal remains may be used for experimentation or other kind of research or study: Yes  N/A
6. No person shall knowingly sell, transfer, distribute or give away any fetus or neonate for a use which is in violation of [Massachusetts General Laws Chapter 112 Section 12J](https://malegislature.gov/Laws/GeneralLaws/PartI/TitleXVI/Chapter112/Section12J): Yes  N/A
7. Individuals engaged in the research will have no part in determining the viability of a neonate:

Yes  N/A

1. For non-viable neonates, the vital functions of the neonate will not be artificially maintained and that the research will not terminate the heartbeat or respiration of the neonate: Yes  N/A
2. For neonates of uncertain viability, the research holds out the prospect of enhancing the probability of survival of the neonate to the point of viability, and any risk is the least possible for achieving that objective: Yes  N/A
3. Can or will subjects who are not yet adults (neonates, children, teenagers) be enrolled?

**Yes  No**

If **Yes**, respond to all of the following:

1. Check to confirm that you will follow “[SOP: Legally Authorized Representatives, Children, and Guardians (HRP-013)](http://viceprovost.tufts.edu/HSCIRB/files/HRP-013_SOP_Legally-Authorized-Representatives-Children-Guardians.pdf)” to determine whether a prospective subject has or has not attained the legal age for consent to treatments or procedures involved in the research under the applicable law of the jurisdiction in which the research will be conducted. (e.g., individuals under the age of 18 years). If this SOP will not be followed, describe how this (attainment of legal age for consent or not) will be determined:
2. How permission to participate in the study will be obtained from the parents or legal guardians:
3. The assent process of children as follows:
   1. Any waiting period available between informing the prospective subject and obtaining the assent:
   2. Any process to ensure ongoing assent:
   3. Research team members involved in the assent process:
   4. How long children will have to consider study participation:
   5. Steps that will be taken to minimize the possibility of coercion or undue influence:
   6. Steps that will be taken to ensure the subjects’ understanding:
   7. If assent will not be obtained from children, specify why:
   8. If children reach 18 years of age while in the study describe the following:
      1. The plan to obtain written informed consent from the subject at age 18 years:
      2. Who will be responsible for managing the plan:
      3. Where the consent discussion will take place:
      4. What will happen if the subject cannot be located to provide consent at age 18 years:
4. Can or will minors who are:
5. married, widowed, divorced; or
6. the parent of a child; or
7. a member of any of the armed forces; or
8. pregnant or believes herself to be pregnant; or
9. living separate and apart from his/her parent or legal guardian, and is managing his/her own financial affairs

be approached for study participation for either themselves or their child?

**Yes  No**

If **Yes**, respond to all of the following:

1. How will it be determined that this population has the capacity to consent for this study. Please note that the circumstance of parenthood, pregnancy, etc. may not mean that the person has the same capacity of an adult who can understand the risks, benefits, and alternatives for indicated care. Thus, sound and sensitive clinical judgment that is attentive to both the minor’s rights and the minor’s actual competence and needs must be considered, and is to include a determination as to whether involvement of family or other adults familiar to the minor is necessary and appropriate:
2. How informed consent will be executed with this population in a way that allows for independent and thoughtful decision-making:
3. Any additional steps or procedures that will be used when performing informed consent with this population:

Refer to Massachusetts state law ([MGL Chapter 112, Section 12F](http://www.malegislature.gov/Laws/GeneralLaws/PartI/TitleXVI/Chapter112/Section12F)) for more information about this population. Please note, the statute applies to clinical care and should be applied to research only as applicable.

1. Can or will wards of the state and/or children at risk of becoming wards of the state be enrolled (this includes foster children or any child that is in state custody)?

**Yes  No**

If **Yes**, respond to all of the following:

1. *Justification for recruiting and enrolling this population:*
2. *Any additional details about the recruitment methods to be used. If the same recruitment methods previously described in the protocol will be used, then state that:*
3. *Any additional details about the informed consent process to be used. If the same informed consent process for enrolling minors previously described in the protocol will be used, then state that:*
4. *How it will be ensured that the appropriate person(s) grants permission for each ward to participate in the research:*
5. *How the research team will know if there has been a change in guardianship status during the course of the research and how permission will be obtained from the new guardian:*
6. *Check to confirm that documentation of review by the Department of Children and Families (DCF) for this study has been submitted to the IRB.*
7. *If the study is greater than minimal risk, describe the following* or  N/A, study is not greater than minimal risk:
8. *Whether the research is related to their status as wards OR if the research is conducted in schools, camps, hospitals, institutions, or similar settings in which the majority of children involved as subjects are not wards:*
9. *How an advocate will be appointed for each child who is a ward, in addition to any other individual acting on behalf of the child as guardian or in loco parentis:*
10. *The background and experience of the advocate to act in the best interests of the child for the duration of the child’s participation in the research:*
11. **☐***Check to confirm that the advocate will not be associated in any way (except in the role as advocate or member of the IRB) with the research, the investigator(s), or the guardian organization.*

Refer to the IRB’s [Wards of the State policy](https://viceprovost.tufts.edu/tufts-health-sciences-irb-policy-wards-state) for additional information on enrolling wards of the state.

1. Can or will cognitively impaired adults (adults with impaired-decision making capacity) or adults who may lose the capacity to consent be enrolled?

**Yes  No**

1. Can or will prisoners be enrolled?

**Yes  No**

If **Yes**, respond to all of the following:

1. *Any possible advantages accruing to the Prisoner through his or her participation in the research, when compared to the general living conditions, medical care, quality of food, amenities and opportunity for earnings in the prison, are not of such a magnitude that his or her ability to weigh the risks of the research against the value of such advantages in the limited choice environment of the prison is impaired:*
2. *Whether the risks involved in the research are commensurate with risks that would be accepted by non-Prisoner volunteers:*
3. *Procedures for the selection of subjects within the prison which are fair to all Prisoners and immune from arbitrary intervention by prison authorities or Prisoners. Unless the Principal Investigator provides to the Board justification in writing for following some other procedures, control subjects must be selected randomly from the group of available Prisoners who meet the characteristics needed for that particular research project:*
4. *Check to confirm that parole boards will not take into account a Prisoner’s participation in the research in making decisions regarding parole, and each Prisoner is clearly informed in advance that participation in the research will have no effect on his or her parole.*
5. *Check to confirm that a letter of support from the Prison this research will be conducted in has been submitted to the IRB.*
6. *If follow-up examination or care of subjects after the end of their participation is required, describe the provision for such examination or care, taking into account the varying lengths of individual Prisoners’ sentences, and for informing subjects of this fact:*
7. Can or will students and/or employees be **targeted** for enrollment in this research?

**Yes  No**

If **Yes**, respond to all of the following:

1. Justification for specifically targeting recruitment efforts to enroll students and/or employees:
2. How potential coercion will be eliminated:
3. Recruitment methods to be applied specifically to students and/or employees. If the same recruitment methods previously described in the protocol will be used, then state that:
4. Additional safeguards included to protect the rights and welfare of students and employees:
5. Protections to ensure that a subject’s decision about participation and/or early withdrawal from the study will not affect his/her status as a student or employee:
6. Check to confirm that you have submitted a letter from the appropriate institutional official (e.g., Department Chair, Dean, Vice-President) who oversees the students and/or employees attesting to the fact that the employee’s or student’s participation in the research is acceptable and that coercion has been minimized.
7. Transgender Subjects: Are you recording sex or gender for your study?

**Yes  No**

Is there a scientific and/or safety rationale for collecting information on whether a subject is transgender?  Yes  No

If **no**, there is no scientific or safety-related rationale for needing to identify sex or gender for your study, and you will not collect information on transgender and gender nonconforming people’s identification.

# Adverse Event Monitoring

## H.1 Definitions

*Define adverse events (AEs), serious adverse events (SAEs), and unanticipated problems for your study:*

The study does not anticipate any adverse events as there is no experimental procedure or intervention being performed.

##

## H.2 Reporting Procedures

1. *Describe the protocol-specific reporting procedures, including who will be responsible for each step (e.g., PI, Data Coordinating Center, Medical Monitor), which forms should be completed, timeframes for reporting, how reports will be distributed, and what follow-up is required: NA*
2. *Include specific details of reporting procedures for:*
   1. *Deaths, life-threatening events, pregnancies: NA*
   2. *Other SAEs: NA*
   3. *Other AEs: NA*
   4. *Other UPs: NA*

Ensure that the reporting procedures meet the reporting requirements of the FDA, NIH, OHRP, sponsor, study leadership and any other regulatory body that applies to the study, as applicable.

##

## H.3 Reportable New Information

*Check to confirm that reportable new information will be reported to the IRB per the Tufts Health Sciences IRB’s* [*Reportable New Information policy.*](https://viceprovost.tufts.edu/reportable-new-information) *If your reporting plan to the IRB differs from the IRB’s policies, please describe it in detail or specify where this information is in the protocol:*

# Statistical Considerations

##

## I.1 Study Endpoints

1. Describe the primary and secondary study endpoints:

The primary outcome of interest is the overall diagnostic accuracy of the automated LVEF, stroke volume, and cardiac output computation as compared to either TTE (for LVEF assessments) or right heart catheterization (for stroke volume and cardiac output assessments).

1. Describe any primary or secondary safety endpoints:

- Time to LVEF acquisition with FoCUS

##

## I.2 Sample Size Justification Statistical Analyses

1. Describe the statistical analyses that will be performed for this study:

All patients who are enrolled will be included in the analysis and the data will be analyzed according to the intention-to-treat principle, with the exception of patients who are found to have moderate or greater regurgitant valvular disease following enrollment. Continuous variables will be summarized as either mean and standard deviation or median and interquartile range, as appropriate. Categorical variables will be summarized by frequency and percentage. The Chi-square or the Fisher’s exact test will be used to compare discrete variables, and the Student’s *t* test or Mann-Whitney U-test will be used to compare continuous variables, as appropriate.

First, the differences between the FoCUS and TTE LEVFs, and the differences between the FoCUS and right heart catheterization stroke volumes and cardiac outputs, will be evaluated for normality bot visually and statistically. This will be done by visual evaluation of quantile-quantile plots, and by applying the Shapiro-Wilk test.^11^ Second, we will evaluate for the presence of proportional bias by performing a regression analysis.^12^ Third, in the case of paired measurements (i.e. if a patient has more than one FoCUS examination performed), we will perform a test for autocorrelation. If the autocorrelation is determined to be non-negligible a correction for the use of paired measurements will be applied.^12^ Finally, the agreement between the FoCUS and TTE LVEF’s will be evaluated through the construction of a Bland-Altman plot along with the mean difference (bias), 95% limits of agreement (LOA), and mean error – along with their corresponding 95% confidence intervals.^13^

1. Provide a sample size justification:

Based on a 95% agreement between FoCUS and formal echocardiography, and a 5% error margin, we anticipate that we would need 73 patients with adequate windows to obtain an AI LVEF. As not all patients may have adequate windows to calculate a biplane method of disks summation (modified Simpson’s rule) and individuals may be found to have moderate or great regurgitant valvular disease following formal TTE, we will aim to recruit 130 patients for the LVEF component of this project.

Based on a 90% agreement between FoCUS and right heart catheterization, and a 10% error margin, we anticipate that we would need 35 patients with adequate windows to obtain an AI stroke volume or cardiac output. As not all patients may have adequate windows to calculate a biplane method of disks summation (modified Simpson’s rule), we will aim to recruit 70 patients for the stroke volume and cardiac output component of this project.

Therefore, in total, we plan to recruit 200 patients for this study.

##

## I.3 Number of Subjects

- 1. Specify the number of subjects to be enrolled in total across all sites:       or  N/A this is not a multicenter study.
  2. Specify the number of subjects to be enrolled at the Tufts site. Subjects who sign an ICF are considered “enrolled”. For studies that have a separate screening ICF, this number is the number of subjects who sign a screening ICF: 200

1. Provide the rationale for enrolling this number of subjects at the Tufts site: See sample size section above.
2. Estimate the number of subjects expected to be enrolled at the Tufts site (i.e. sign the screening or study ICF) as well as the number needed to complete the study at the Tufts site: See sample size justification above.
3. If a large number of withdrawals and/or dropouts is expected, explain why: See sample size justification above.

##

## I.4 Data Management

- 1. Describe the data analysis plan, including descriptions of the data: See above section on statistical analysis
  2. Describe the steps that will be taken to secure the data (e.g., training, authorization of access, password protection, encryption, physical controls, certificates of confidentiality, and separation of identifiers and data) during storage, use, and transmission:

Personal health information will be collected and used by the Principal Investigator and his authorized co-investigators as part of this research protocol. All information collected throughout the study will be kept strictly confidential and only entered into the RedCap secure data server. For purposes of analysis all data extracted from the RedCap server will be de-identified. The de-identified data will be entered into a password protected spreadsheet stored on a secure hospital server. Subjects will only be identified by a unique ID. No identifiable information will be stored on any mobile devices. There will be no patient interventions performed for this study. All data will be kept and stored securely as per regulation

- 1. Describe any procedures that will be used for quality control of collected data: NA

## I.5 Randomization

Will subjects be randomized?

**Yes  No**

# Drugs or Devices

- 1. Will the research involve drugs?

**Yes  No**

- 1. Will the research involve devices?

**Yes  No**

If **Yes**, respond to all of the following:

1. If the device has an IDE or a claim of abbreviated IDE (non-significant risk device) identify the holder of the IDE/Abbreviated IDE:      The device used in this study is an FDA approved device. Please see submitted documentation in IRB application.
2. Describe your plans to store, handle, and administer study devices so that they will be used only on subjects and be used only by authorized investigators (if the control of the devices used in this protocol will be accomplished by following an established, approved organizational SOP, please reference that SOP in this section):      The device used in this study is approved for clinical use by the FDA. There is no reason that it can not be used for non-research purposes by the investigators. As described in detail above, if the device is to be used for research purposes all of the aforementioned protocols and informed consent process will be followed prior to using the device for research purposes.
3. Specify who will be responsible for the costs of implantation or placement of the device in subjects’ bodies?:       or N/A, the device will not be implanted or placed in the body.
4. Specify who will be responsible for the costs of removing the device from subjects’ bodies?       or N/A, the device will not be removed from the body.
5. Specify the cost of the device and who will be responsible for the cost:       or N/A, the device will be provided free of charge.
6. Who on the research team, in addition to the Principal Investigator, will be accountable for device(s):      The co-investigators
7. Who will interface with the sponsor:      There is no study sponsor
8. The study device or procedure (including beneficial health care procedures) will be available to subjects after participation in the study: Yes N/A
9. Handouts or instructions sheets that will be given to subjects on how to use study device(s) have been submitted to the IRB: Yes N/A

# Study Administration

## K.1 Setting

1. Describe the sites / locations where your research team will conduct the research: Tufts Medical Center
2. The research will take place at an international site, and the [International Research Guidance](https://eirb.tuftsmedicalcenter.org/IRB/sd/Doc/0/48BQMF8656K4P76QU0ERKMDL6A/International%20Research%20Guidance_Downloaded%20from%20IRB%20website_09.06.19.docx) and [International Checklist](https://eirb.tuftsmedicalcenter.org/IRB/sd/Doc/0/4FUMCVJNRQF4T54J3AVET8NU33/International%20Research%20Checklist_071519.docx) were utilized:  Yes  N/A

## K.2 Registration

1. *Describe the steps the research team will take to ensure that a subject is appropriately enrolled or registered in the study prior to receiving any study intervention (e.g. describe and submit any protocol eligibility checklist that will be used, specify who on the research team will confirm eligibility and that consent was documented, etc.):* As noted above the investigator and co-investigators will determine subject eligibility through review of the echocardiography and right heart catheterization schedules. Once patients are identified the patients will be approached regarding their interest in participation in the study. The step by step procedures to be followed have been outlined in the informed consent form.

## K.3 Resources Available

1. Describe the roles/tasks of each research team member here (or alternatively, you may submit any current Delegation of Authority Log you may have which already has this information completed):

All research team members will participate in subject screening, enrollment, consent, data collection, and performing bedside ultrasound examinations.

1. Describe the qualifications (e.g., training, experience) of the PI and research team to perform their roles. Provide enough information for the IRB to determine the PI and research team are qualified to conduct the proposed research. Alternatively, you can submit the current CVs for the research team instead:

Dr. Huggins is a board certified cardiologist and an Associate professor of medicine at Tufts University with an extensive background in clinical and non-clinical research. Drs. Marbach and Hon are board certified in cardiology and pulmonology/critical care, respectively, and are currently completing advanced clinical fellowship training at Tufts, They have both been involved in the design and implementation of multiple retrospective and prospective clinical studies.

1. Describe the coverage plan to address any issues (including subject safety issues) that occur while the PI is away and/or unavailable. The research team member designated to serve as the acting PI in the PI’s absence should have similar training and expertise as the PI: Dr. Marbach will serve as the acting PI should any issues arise when Dr. Huggins is unavailable.
2. Describe the process to ensure the research team members have adequate oversight and are adequately trained regarding the protocol, study procedures, and their roles and responsibilities: The research team includes the investigator and two co-investigators. All three members of this team participated in developing the research question and protocol. They are all fully aware of the research protocol, study procedure, and their roles. If any amendments to the protocol are made all three members of the research team will be informed prior to said amendments. All research team members have confirmed their participation in the study and have agreed to remain up-to-date with any changes in the study protocols and/or procedures.
3. Medical or psychological resources that subjects might need, such as for emergencies or medical issues, are available for the study: Yes  N/A

##

## K.4 IRB Review

1. *Check to confirm that an appropriate IRB registered with the OHRP, will review and approve this study.*
2. *Check to confirm that any amendments to the protocol or informed consent documents will be reviewed and approved by the IRB prior to use, unless required to eliminate an apparent immediate hazard to subjects.*

## K.5 Multi-Site Research

Is this a multi-site study where Tufts is the sponsor, primary grant recipient, or coordinating site?:

**Yes  No**

## K.6 Community-Based Participatory Research

Can or will this study involve community-based participatory research?

**Yes  No**

If **Yes**, respond to all of the following

1. *Describe the communities that will be involved in this research:*
2. Describe the composition and involvement of any community advisory board:
3. *Describe the involvement of the community in the design, protocol development, informed consent process, access to data and samples, and conduct of the research*
4. *Describe the plans on dissemination and publication of study results which are in agreement with the community:*

## K.7 Sharing Results with Subjects

Will results (overall study results or individual subject results, such as results of investigational diagnostic tests, genetic tests, or incidental findings) be shared with subjects or others (e.g., the subject’s primary care physician or the subject’s treating physician)?

**Yes  No**

If **Yes**, respond to all of the following:

1. Rationale for sharing these results:
2. How results will be shared:
3. For individual subject results, specify if subjects have the option to opt-in or opt-out of receiving these results or allowing these results to be shared with others:
4. *Any referral policies (i.e. for confirmation of any individual subject results):*
5. *Check to confirm that testing of research specimens is being conducted in a laboratory certified (CLIA-approved) to conduct diagnostic testing. If patient-specific research results are reported from the laboratory and those results will or could be used for the diagnosis, prevention, or treatment of any disease or impairment, or the assessment of the health of human beings, the laboratory must be* [*CLIA certified*](https://www.cms.gov/Regulations-and-Guidance/Legislation/CLIA/Downloads/Research-Testing-and-CLIA.pdf).
6. *If the research tests are experimental or of unknown or unproven clinical significance and the results will be provided to the source individual or physician or placed in the source individual’s medical record, provide the rationale for this:*

#

# References

1. Mirabel M, Celermajer D, Beraud A-S, Jouven X, Marijon E, Hagège AA. Pocket-sized focused cardiac ultrasound: Strengths and limitations. *Arch Cardiovasc Dis* [Internet]. 2015 [cited 2018 Sep 26];108:197–205. Available from: https://linkinghub.elsevier.com/retrieve/pii/S1875213615000108

2. Marbach JA, Almufleh A, Di Santo P, Jung R, Simard T, McInnes M, Salameh J-P, McGrath TA, Millington SJ, Diemer G, West FM, Domecq M-C, Hibbert B. Comparative Accuracy of Focused Cardiac Ultrasonography and Clinical Examination for Left Ventricular Dysfunction and Valvular Heart Disease: A Systematic Review and Meta-analysis. *Ann Intern Med* [Internet]. 2019 [cited 2020 Jan 20];171:264. Available from: https://annals.org/aim/fullarticle/2747507/comparative-accuracy-focused-cardiac-ultrasonography-clinical-examination-left-ventricular-dysfunction

3. Marbach JA, Almufleh A, Santo PD, Simard T, Jung R, Diemer G, West FM, Millington SJ, Mathew R, May MRL, Hibbert B. A Shifting Paradigm – The Role of Focused Cardiac Ultrasound in Bedside Patient Assessment. *CHEST* [Internet]. 2020 [cited 2020 Aug 27];0. Available from: https://journal.chestnet.org/article/S0012-3692(20)31950-4/abstract

4. Martindale JL, Wakai A, Collins SP, Levy PD, Diercks D, Hiestand BC, Fermann GJ, deSouza I, Sinert R. Diagnosing Acute Heart Failure in the Emergency Department: A Systematic Review and Meta-analysis. *Acad Emerg Med* [Internet]. 2016 [cited 2018 Oct 3];23:223–242. Available from: http://doi.wiley.com/10.1111/acem.12878

5. Spencer KT, Kimura BJ, Korcarz CE, Pellikka PA, Rahko PS, Siegel RJ. Focused Cardiac Ultrasound: Recommendations from the American Society of Echocardiography. *J Am Soc Echocardiogr* [Internet]. 2013 [cited 2018 Sep 26];26:567–581. Available from: http://linkinghub.elsevier.com/retrieve/pii/S0894731713002599

6. Labovitz AJ, Noble VE, Bierig M, Goldstein SA, Jones R, Kort S, Porter TR, Spencer KT, Tayal VS, Wei K. Focused Cardiac Ultrasound in the Emergent Setting: A Consensus Statement of the American Society of Echocardiography and American College of Emergency Physicians. *J Am Soc Echocardiogr* [Internet]. 2010 [cited 2018 Oct 1];23:1225–1230. Available from: http://linkinghub.elsevier.com/retrieve/pii/S0894731710008710

7. Mayo PH, Beaulieu Y, Doelken P, Feller-Kopman D, Harrod C, Kaplan A, Oropello J, Vieillard-Baron A, Axler O, Lichtenstein D, Maury E, Slama M, Vignon P. American College of Chest Physicians/La Société de Réanimation de Langue Française Statement on Competence in Critical Care Ultrasonography. *Chest* [Internet]. 2009 [cited 2018 Oct 1];135:1050–1060. Available from: http://linkinghub.elsevier.com/retrieve/pii/S0012369209602609

8. Arntfield RT, Millington SJ, Ainsworth CD, Arora RC, Boyd J, Finlayson G, Gallagher W, Gebhardt C, Goffi A, Hockmann E, Kirkpatrick AW, McDermid RC, Waechter J, Wong N, Zavalkoff S, Beaulieu Y. Canadian Recommendations for Critical Care Ultrasound Training and Competency. *Can Respir J* [Internet]. 2014 [cited 2018 Oct 15];21:341–345. Available from: http://www.hindawi.com/journals/crj/2014/216591/

9. Lang RM, Badano LP, Mor-Avi V, Afilalo J, Armstrong A, Ernande L, Flachskampf FA, Foster E, Goldstein SA, Kuznetsova T, Lancellotti P, Muraru D, Picard MH, Rietzschel ER, Rudski L, Spencer KT, Tsang W, Voigt J-U. Recommendations for Cardiac Chamber Quantification by Echocardiography in Adults: An Update from the American Society of Echocardiography and the European Association of Cardiovascular Imaging. *J Am Soc Echocardiogr* [Internet]. 2015 [cited 2019 Jan 30];28:1-39.e14. Available from: https://linkinghub.elsevier.com/retrieve/pii/S0894731714007457

10. Dadon Z, Rosenmann D, Butnaru A, Glikson M, Alper-Suissa L, Alpert EA. Use of Artificial Intelligence by Medical Students to Enable Accurate Point-of-Care Echocardiographic Assessment of Ejection Fraction. *J Am Coll Cardiol* [Internet]. 2020 [cited 2020 Aug 27];75:1568. Available from: https://www.onlinejacc.org/content/75/11_Supplement_1/1568

11. Razali NM, Wah YB. Power comparisons of Shapiro-Wilk, Kolmogorov-Smirnov, Lilliefors and Anderson-Darling tests. :14.

12. Bland JM, Altman DG. Measuring agreement in method comparison studies. *Stat Methods Med Res*. 1999;8:135–160.

13. Gerke O. Reporting Standards for a Bland–Altman Agreement Analysis: A Review of Methodological Reviews. *Diagnostics* [Internet]. 2020 [cited 2020 Aug 28];10. Available from: https://www.ncbi.nlm.nih.gov/pmc/articles/PMC7278016/

# M. Protocol Ammendment

Once all study activities have been finalized, we will combine our study results with those from a similar study performed at an outside institution.

As described in detail above, our study evaluated the accuracy of the KOSMOS artificial intelligence generated ejection fraction compared to formal echocardiography. Another institution (Ottawa Heart Institute) has performed a similar study with the same device. We have agreed to combine our non-identifiable data so that the results of our studies will be more generalizable across institutions and in order to increase the power of our results.

As noted above, no identifiable information will be shared. The following variables will be shared:

- demographics: sex, age, height, weight

- past medical history: diabetes, dyslipidemia, prior MI, prior PCI, prior CABG, prior stroke, heart failure, COPD, hypertension, smoking history

- KOSMOS results: ejection fraction, stroke volume

- formal echocardiogram results: need for echo contrast, formal ejection fraction, presence or absence of valvular heart disease
