# Supplementary material for: Diagnostic accuracy of point-of-care ultrasound with artificial intelligence-assisted assessment of left ventricular ejection fraction
Source: NPJ Digit Med. 2023 Oct 28;6:201. doi: 10.1038/s41746-023-00945-1 (PMC10613290; doi:10.1038/s41746-023-00945-1)
Supplement: Supplementary file 4 — Reporting Summary [file 41746_2023_945_MOESM4_ESM.pdf]

nature portfolio

Corresponding author(s): Dr Benjamin Hibbert

Last updated by author(s): Aug 30, 2023

Reporting Summary

Nature Portfolio wishes to improve the reproducibility of the work that we publish. This form provides structure for consistency and transparency in reporting. For further information on Nature Portfolio policies, see our Editorial Policies and the Editorial Policy Checklist.

Statistics

For all statistical analyses, confirm that the following items are present in the figure legend, table legend, main text, or Methods section.

n/a	Confirmed	
☐	☒	The exact sample size (*n*) for each experimental group/condition, given as a discrete number and unit of measurement
☒	☐	A statement on whether measurements were taken from distinct samples or whether the same sample was measured repeatedly
☐	☒	The statistical test(s) used AND whether they are one- or two-sided<br>*Only common tests should be described solely by name; describe more complex techniques in the Methods section.*
☐	☒	A description of all covariates tested
☒	☐	A description of any assumptions or corrections, such as tests of normality and adjustment for multiple comparisons
☐	☒	A full description of the statistical parameters including central tendency (e.g. means) or other basic estimates (e.g. regression coefficient) AND variation (e.g. standard deviation) or associated estimates of uncertainty (e.g. confidence intervals)
☐	☒	For null hypothesis testing, the test statistic (e.g. *F*, *t*, *r*) with confidence intervals, effect sizes, degrees of freedom and *P* value noted<br>*Give P values as exact values whenever suitable.*
☒	☐	For Bayesian analysis, information on the choice of priors and Markov chain Monte Carlo settings
☒	☐	For hierarchical and complex designs, identification of the appropriate level for tests and full reporting of outcomes
☒	☐	Estimates of effect sizes (e.g. Cohen's *d*, Pearson's *r*), indicating how they were calculated

Our web collection on statistics for biologists contains articles on many of the points above.

Software and code

Policy information about availability of computer code

Data collection	Data was manually collected and stored on the REDCap software.
Data analysis	Analyses were performed using SAS software, version 9.4 (SAS Institute, North Carolina, USA).

For manuscripts utilizing custom algorithms or software that are central to the research but not yet described in published literature, software must be made available to editors and reviewers. We strongly encourage code deposition in a community repository (e.g. GitHub). See the Nature Portfolio guidelines for submitting code & software for further information.

Data

Policy information about availability of data

All manuscripts must include a data availability statement. This statement should provide the following information, where applicable:

- Accession codes, unique identifiers, or web links for publicly available datasets
- A description of any restrictions on data availability
- For clinical datasets or third party data, please ensure that the statement adheres to our policy

Due to the strict ethical approvals provided in this study, the data used in this study is available from the corresponding author upon request and after establishment of data sharing agreement between institutions.

Research involving human participants, their data, or biological material

Policy information about studies with human participants or human data. See also policy information about sex, gender (identity/presentation), and sexual orientation and race, ethnicity and racism.

Reporting on sex and gender	The overall number of males and females are highlighted in table 1. As an observational study, using patient electronic records, we were unable to confirm gender.<br><br>Sex-specific findings were not calculated as the purpose of this study was to compare bedside ultrasound findings to formal echocardiography.
Reporting on race, ethnicity, or other socially relevant groupings	We did not complete race, ethnicity or socially relevant groupings sub-analyses.
Population characteristics	Please refer to Table 1. We highlighted patient demographics (age, sex, BMI), previous medical history and echocardiography findings.
Recruitment	We recruited patients using convenience sampling. Sampling bias is a known disadvantage with this type of study design, as well as researcher bias. We attempted to recruit patients from various settings (inpatient ward, ICU, outpatient) to reduce the impact of sampling bias.
Ethics oversight	Ethics approval was obtained from the Ottawa Health Science Network Research Ethics Board and Tufts Institutional Review Board (Supplementary Document).

Note that full information on the approval of the study protocol must also be provided in the manuscript.

Field-specific reporting

Please select the one below that is the best fit for your research. If you are not sure, read the appropriate sections before making your selection.

☒ Life sciences ☐ Behavioural & social sciences ☐ Ecological, evolutionary & environmental sciences

For a reference copy of the document with all sections, see nature.com/documents/nr-reporting-summary-flat.pdf

Life sciences study design

All studies must disclose on these points even when the disclosure is negative.

Sample size	There was no a prior sample size calculation. Patients were prospectively recruited for 16 months, which we thought would allow for a sufficient sample size. At a sample size of 449 participants, we believed we had sample size for our objectives.
Data exclusions	Recruitment: There were no exclusion criteria.<br>Data Analysis: Studies were excluded from the data analysis if the images were non-diagnostic, and the device was unable to calculate a LVEF.
Replication	Replication does not apply to our study design.
Randomization	As this was an observational study, this does not apply.
Blinding	As this was an observational study, this does not apply.

Reporting for specific materials, systems and methods

We require information from authors about some types of materials, experimental systems and methods used in many studies. Here, indicate whether each material, system or method listed is relevant to your study. If you are not sure if a list item applies to your research, read the appropriate section before selecting a response.

Materials & experimental systems

n/a	Involved in the study
☒	☐ Antibodies
☒	☐ Eukaryotic cell lines
☒	☐ Palaeontology and archaeology
☒	☐ Animals and other organisms
☐	☒ Clinical data
☒	☐ Dual use research of concern
☒	☐ Plants

Methods

n/a	Involved in the study
☒	☐ ChIP-seq
☒	☐ Flow cytometry
☒	☐ MRI-based neuroimaging

Clinical data

Policy information about clinical studies

All manuscripts should comply with the ICMJE guidelines for publication of clinical research and a completed CONSORT checklist must be included with all submissions.

Clinical trial registration: This is an observational study and was therefore not registered on clinicaltrials.gov.

Study protocol: The study protocol/ethics submission has been included as a supplementary text.

Data collection: Data collection was completed by extracting FoCUS findings from the ultrasound tablets, and patient demographics and echocardiography findings were extracted from their electronic chart.

Outcomes: The objectives were highlighted in our protocol/ethics submission, which has been included as a supplementary text.
